# Artichoke Industrial Waste in Durum Wheat Bread: Effects of Two Different Preparation and Drying Methods of Flours and Evaluation of Quality Parameters during Short Storage

**DOI:** 10.3390/foods12183419

**Published:** 2023-09-14

**Authors:** Michele Canale, Rosalia Sanfilippo, Maria Concetta Strano, Margherita Amenta, Maria Allegra, Ilaria Proetto, Martina Papa, Rosa Palmeri, Aldo Todaro, Alfio Spina

**Affiliations:** 1 Council for Agricultural Research and Economics (CREA), Research Centre for Cereal and Industrial Crops, Corso Savoia, 190, 95024 Acireale, Italy; rosalia.sanfilippo@crea.gov.it; 2 Council for Agricultural Research and Economics (CREA), Research Centre for Olive, Fruit and Citrus Crops, Corso Savoia, 190, 95024 Acireale, Italy; mariaconcetta.strano@crea.gov.it (M.C.S.); margherita.amenta@crea.gov.it (M.A.); maria.allegra@crea.gov.it (M.A.); martina.papa@crea.gov.it (M.P.); 3 Department of Agriculture, Food, Environment (Di3A), University of Catania, Via S. Sofia, 98, 95123 Catania, Italy; ilaria.proetto@phd.unict.it (I.P.); rpalmeri@unict.it (R.P.); 4 DSAAF—Dipartimento di Scienze Agrarie, Alimentari e Forestali, University of Palermo, Viale delle Scienze, 12 Ed. 4, 90128 Palermo, Italy; aldo.todaro@unipa.it

**Keywords:** artichoke flour, *Cynara scolymus*, durum wheat, functional bread, polyphenols, staling process, sustainability, texture profile analysis, upcycling

## Abstract

‘Violetto di Ramacca’ is a local variety of artichoke grown in Sicily (Southern Italy), known for its purple color with green streaks. In this study, the effects of two different preparation and drying methods (method A, fresh sample oven-dried at 40 °C for 48 h then mixed and ground into flour; and B, minced and frozen sample oven-dried at 40 °C for 24 h then blended and ground into flour) for flours from different parts of the artichoke (bracts, stems, and mix), used at different percentages of integration (5, 7.5, and 10%), in combination with re-milled semolina, have been evaluated. The polyphenol contents of the flours produced with the two methods were measured. The results showed significant differences between the methods and samples, with a range from 9.09 mg GAE/g d.m. (bracts 100%, method A) to 2.62 mg/g (mix 100%, method B). The values were then lowered in the flour products with supplements ranging from 0.96 mg GAE/g (bract flour 10%, method A) to 0.11 mg GAE/g (mixed flour 7.5%, method B). As the amounts of polyphenols increased, the antioxidant activity increased, with a range that varied in the pure flour from 8.59 mg trolox eq/g d.m. (bract flour, method A) to 3.83 mg trolox eq/g d.m. (mixed flour, method B). These flours were also analyzed for color, highlighting a clear difference between methods A (greener) and B (browner). The flours thus obtained were used to produce breads, which were evaluated for their physicochemical characteristics during 4 days of storage. The results showed a reduction in volumes and heights, an increase in the percentage of integration of the artichoke flours, a greater quantity of moisture in the integrated breads, and a lower reduction in the structural characteristics during storage compared to the control breads. The TPA was conducted on the breads from T_0_ to T_4_, highlighting that, although initially more compact, the integrated breads offered less alteration of the values during storage. The a_w_ ranged from 0.63 (mix flour 5%, method B) to 0.90 (bract flour 5%, method B). The amounts of polyphenols (from 0.57 mg GAE/g in bread with bracts at 10% (method A) to 0.13 mg GAE/g in bread with mix 5% (method B)) and the antioxidant activity (from 0.55 mg trolox eq/g d.m. in bread with bract flour 10% (method A) to 0.14% mg trolox eq/g d.m. in bread with mix flour) were also evaluated, showing a trend similar to the values obtained in the flours. Colorimetric tests highlighted a color more similar to wholemeal bread in the loaves produced with method B. Statistical factor analysis and cluster analysis were conducted for all trials.

## 1. Introduction

In the vast panorama of plants widespread in the Mediterranean area, the artichoke (*Cynara cardunculus* subsp. *scolymus* (L.) Hayek) represents an important food from an economic point of view [1]; especially in Italy, it is one of the most important horticultural crops, together with tomatoes and potatoes [2].

It is a perennial herbaceous plant belonging to the Asteraceae family [3], characterized by an edible inflorescence known as a ‘flower head’, fleshy leaves, bracts, and a receptacle, which is also edible [2], and which represent about 30–40% of the weight of the plant [4].

The artichoke is also widely appreciated for its nutritional profile thanks to the presence of minerals, fibers, and polyphenolic compounds. Furthermore, there is a hypoglycemic oligosaccharide called inulin which can be used as a sugar substitute for diabetic subjects [5,6], with a positive effect on the intestinal microflora [7].

The artichoke, in particular, contains mono- and dicaffeoylquinic acids (chlorogenic acid and cynarin) and flavonoids (luteolin, apigenin, and their glucosides and rutinosides) with an antioxidant action. The highest concentration is found mainly in the leaves and flower heads [8]. Specifically, during plant development, phenolic compounds tend to accumulate in the peripheral parts of the plant, where they perform their biological functions [9,10]. When compared to other vegetables, artichoke flower heads have higher contents of total polyphenols [11].

Humans cannot synthesize these compounds; therefore, it is necessary to take them through food [12]. Bread is a very important food in people’s diet [13]; therefore, it is widely used to convey functional components useful to man [14,15].

In recent years, moreover, there has been a growing interest in the reuse of agrifood waste [10] to produce new products as a sustainable means of development [16,17].

The increase in world population is inevitably leading to greater waste production, with harmful effects on the environment. Research is particularly oriented towards these issues, focusing on the possibility of developing agrifood systems in order to minimize the environmental impact and waste of resources [18,19,20]. A key action in achieving this is the recovery and reuse of agrifood processing waste, which is often rich in functional compounds that allow it to be transformed into resources for new food products.

The artichoke is one of the crops most prone to waste production during industrial processing. Approximately 80% of the total plant biomass of the artichoke consists of bracts and stems, which are discarded because they are unsuitable for human consumption [21]. Therefore, they become waste. However, the presence of substances with high nutritional value potentially makes these components suitable for the production of enriched foods.

The industrial treatment of bracts and stems, through a preliminary phase of drying and subsequent milling, makes it possible to obtain a flour that can be blended with re-milled semolina for the production of durum wheat bread.

The aim of this work was to evaluate the impact of two different methods of preparing artichoke waste flour (bracts and stems) on the polyphenol profile and antioxidant activity of the flours and bread produced, as well as on some physicochemical parameters such as moisture color and water activity. In addition, the physical characteristics of the loaves and their behavior during a storage period of 4 days were evaluated in order to evaluate the bread staling process.

## 2. Materials and Methods

### 2.1. Materials

Sicilian durum wheat, re-milled semolina, from the agricultural cooperative society ‘Valle del Dittaino’ a.r.l. in Assoro (Enna, Italy), certified for ‘Dittaino PDO bread’, was used for the tests, to which bract flour, stem flour, and their mix (50–50) were added at different percentages (5.0%, 7.5%, 10.0%, respectively) [8].

The flour was obtained via the method described below from artichoke (*Cynara cardunculus* subsp. *scolymus* (L.) Hayek), cv. ‘Violetto di Ramacca’, following the industrial processing of the flower heads, which involves the removal of the outer bracts and stems.

### 2.2. Preparation of Flour from Artichoke

The stems and bracts of artichoke cv. ‘Violetto di Ramacca’ were separated from the hearts to simulate the waste produced by the artichoke canning industry. Only the ex-ternal bracts were taken (about 20–25 for each artichoke). The stems were cut in 1.5 cm long pieces [7].

The flour used was previously obtained from the stems and bracts through two different types of drying, in agreement with the process described by Borsini et al. [22].

As described by Ruiz-Cano et al. [23], the bracts and stems were separated from the hearts of the flower heads by taking 20–25 bracts from each artichoke, while the stems were cut transversely to obtain homogeneous cylindrical portions of 15.0 ± 0.5 mm in length.

Samples were dried using two different methods:

- Fresh sample (A): drying in an oven (Memmert, Milan, Italy) at 40 °C for 48 h. Afterwards, the samples were reduced to smaller fragments using a benchtop blender and then the ‘Cyclotec’ type 120 mill (Falling Number, Huddinge, Sweden) further reduced the flour to diameter of 500 μ.
- Shredded and frozen sample (B): Samples were frozen at −15 °C in a domestic freezer (Beko, Milan, Italy) and then oven-dried (Memmert, Milan, Italy) at 40 °C for 24 h. Then, the samples were cut into smaller fragments using a benchtop blender and reduced to flour with a diameter of 500 μ via the Cyclotec type 120 mill (Falling Number, Huddinge, Sweden). For each drying experiment, the samples reached a final moisture content of between 4 and 6% [24].

The mixes were produced with stem and bract flour in a 1:1 ratio.

### 2.3. Color Determination

The CR 200 Minolta Colorimeter Chroma (Minolta, Osaka, Japan) [25] was used for color evaluation, which was followed by the implementation of the CIELab colorimetric model [26]. L*, a*, and b* coordinates were used to express the results [27].

The brown index, indicating the browning tendency, ranging from 0 to 100, the red index a*, indicating the variation from red to green, and the yellow index b*, indicating the variation from yellow to blue, were determined [28].

The analyses were carried out in triplicate.

### 2.4. Polyphenols Content and Antioxidant Activity (DPPH)

In order to analyze the total polyphenol content, water extracts of each bread sample were prepared according to the method of Parafati et al. [29]. Total polyphenols content was evaluated following a modified Vasquez-Roncero et al. [30] method. Briefly, an aliquot of extract (250 µL) was mixed with Folin–Ciocalteau reagent (1.25 mL); after 3 min of incubation, 20% sodium carbonate (2.5 mL) was added. Finally, the solution was brought to 25 mL. After an incubation of 1 h in the dark, the absorbance at 725 nm was measured with a Perkin Elmer lambda 25 Ultraviolet–Visible spectrometer (PerkinElmer Inc, Waltham, WA, USA). Total polyphenol content was expressed as mg of gallic acid equivalents (GAE)/g of dry matter (DM). The standard curve was acquired with eleven gallic acid concentrations (0–80 mg/mL).

The antioxidant activity of the bread samples was measured with 2,2-diphenyl-1-picrylhydrazyl (DPPH) via the radical scavenging activity method described by Brand-Williams et al. [31] with some modifications. An amount of 50 µL of extract was mixed with 3 mL of DPPH solution, homogenized, and put in dark for 1 h. After the incubation time, absorbance at 515 nm was measured. Antioxidant activity was expressed as mg of trolox equivalent (TE)/kg of dry matter (d.m.) using a standard curve constructed of eight different concentrations (0–75 mg/L).

The analyses were carried out in triplicate.

### 2.5. Physical Characteristics of Breads with Different Integration of Artichoke Flour

In Table 1, the ingredients to produce the different breads are listed. 

Several solutions were prepared in the following way, using the corrected amount of water per 500 Brabender Unit (B.U.):

- Yeast solution: 600 mL distilled water and 80 g yeast (Despar Italia from ZEUS IBA srl, Casalecchio di Reno, Bologna, Italy);
- Salt–sugar solution: 600 g distilled water, 80 g salt (Italkali spa, Palermo, Italy), and 40 g sugar (Conad, Pontelongo, Padua, Italy);
- Ascorbic acid solution: 500 mL distilled water and 0.04 g ascorbic acid (Bontà Infinite srl, Terme Vigliatore, Messina, Italy).

The added distilled water was calculated on the basis farinograph the water absorption [8] by subtracting the distilled water already added with the three solutions.

The dough was left to rise in a thermostatic chamber (Giorik, Sedico, Belluno, Italy) equipped with a steam humidifier (SD/SD series, Carel, Brugine, Padua, Italy) at 30–32 °C, 70–75% RH for 1 h and 30 min in metal molds (7 cm wide, 12.5 cm long); this was followed by baking in an electric oven (Giorik, Sedico, Belluno, Italy) at 170 ± 5 °C for 20 min.

Physical analyses of properties such as volume, height, weight, moisture, crumb porosity, crumb and crust color, texture, and water activity were conducted on the loaves obtained according to the two methods (A and B) described.

The volume of the bread was determined via the rape seed displacement method according to the AACC 10–05 method [32].

The loaf height was measured using a digital caliper (Digi-MaxTM, SciencewareR, NY, USA).

Bread moisture was recorded, following AOAC method 935.25 [33], by drying the bread in a Memmert oven at 103 °C to constant weight. The results were expressed as percentage relative humidity (RH%) [34].

For crumb porosity, central bread slices from each loaf were visually compared with the eight Dallmann reference pictures [35], representing a cross section of breads with different crumb structures. The crumb porosity was evaluated on the basis of the 8-degree Mohs scale as modified by Dallmann [36], where, for mold breads, 1 indicates non-uniform structure (i.e., with large and irregular cells), and 8 indicates uniform compact structure (i.e., we small and regular cells) [8].

Bread crusts’ hardness was assessed by using a texture analyzer (Zwick Z 0.5 Röell, Ulm, Germany) equipped with an 8 mm diameter stainless steel cylindrical flat probe at a test speed of 1 mm/s and with 20% applied deformation (force shutdown threshold). The resulting crust breakdown point was measured in Newton (N).

The other bread texture parameters were carried out on slices (15 mm thickness) using the TPA (texture profile analysis) test according to method of Różylo et al. [37] with slight modifications. The TPA test was performed using the texture analyzer (Zwick Z 0.5 Röell, Ulm, Germany) equipped with a 75 mm diameter stainless steel compression platen probe, which involved double compression to a depth of 50% and 10%, respectively, at a speed of 1 mm/s.

The water activity (Aw) on each sample was measured throough the hygrometric method, at 20 °C via Aqualab Vapor Sorption (Decago Device, Pullman, WA, USA).

Crumb and crust color were assessed as described above for flours.

The analyses were carried out in triplicate.

### 2.6. Determination of Bread Staling Rate

Bread was stored for 4 days at 25 °C, packed in cardboard. On the day of baking, 2 days and 4 days later, the loaf hardness and moisture were determined to evaluate the staling rate according to AACC method 10–10.03 [32]. Loaf hardness and moisture were assessed as described above.

The analyses were carried out in triplicate.

### 2.7. Statistical Analyses

#### 2.7.1. One-Factor and Two-Factor ANOVA

The statistical analysis was performed using the Statgraphics^®^ Centurion XVI software package (Statpoint Technologies, INC., The Plains, VA, USA). One-factor and two-factor analysis of variance (ANOVA), followed by Tukey’s HSD test (*p* ≤ 0.001), was carried out on all physicochemical, technological, and breadmaking attributes and the bread staling process. Three factors were considered: sample, percentage, and method. A one-factor analysis determined the interaction of the factors studied, while a two-factor analysis analyzed each factor’s influence (or lack of influence) individually.

#### 2.7.2. Cluster Analysis

A sequence of two cluster analyses (the hierarchical cluster analysis and the K-means cluster analysis) was conducted on the sets of all the variables studied relating to flours and breads.

Hierarchical cluster analysis was performed as a first step to identify useful patterns and the number of clusters within the large dataset without a priori information.

The K-means cluster analysis was applied in a second step, using the number of clusters extracted from the hierarchical cluster analysis as input for its algorithm, with the aim of assigning and interpreting cluster membership.

To neutralize the impact of variables with large values versus variables with small values on distance measurements, the clustering procedure was preceded by the calculation of standardized scores for the variables, saved as new z-score-variables. Pretreatment for data standardization resulted in all variables contributing equally to the distance measurements.

The statistical analysis was conducted with the IBM SPSS Statistics software package, version 20 (IBM Corporation, 2011, Armonk, NY, USA).

## 3. Results and Discussion

### 3.1. Flours

#### 3.1.1. Polyphenols Content and Antioxidant Activity in Flours

The total polyphenols contents and antioxidant activities, according to the DPPH assay, are shown in Table 2.

Results for total FC (Folin–Ciocalteu reducing capacity), reported as polyphenols (mg of GAE/g d.m.), showed values ranging from 9.09 mg of GAE/g d.m. in FAB (method A) to 0.05 mg GAE/g d.m. in SC, while DPPH values range from 8.59 (FAB, method A) to 0.05 (SC) mg rolox eq/g d.m.

The correlation level between the phenolic content and antioxidant activity between the plant organs is an interesting aspect of this study, which supports the hypothesis that the former compounds contribute directly to antioxidant activity [38].

In this study, the correlation coefficient between Folin–Ciocalteu reducing capacity results (FC) and the IC_50_ values of the DPPH^⋅^ quenching activity was highly significant (*p* < 0.001), indicating that polyphenolics may play an important role in free radical scavenging [39].

The relationship between the antioxidant activity and phenolic compounds depends on numerous factors, such as the chemical structure of individual component, the synergistic interaction among them, and the specific conditions applied in different assays [40,41].

In the specific case of the polyphenols content, it is possible to notice a statistical difference between the pure flours made with methods A and B (Supplementary Table S1), both with regard to the flours produced with the various parts of the artichoke (Supplementary Table S2) and for the different integrated percentages (Supplementary Table S3).

Artichoke bracts flour is richer in polyphenols than artichoke stems flours, whereas the polyphenols content of the fresh product was 8 mg GAE/g [42], in agreement with other authors [43].

Moreover, as regards the artichoke bract flours, it was possible to observe that method B caused their enzymatic oxidation, catalyzed by polyphenol oxidase (PPO) [44], and therefore a reduction of the polyphenols content and the typical browning [45,46].

#### 3.1.2. Color Parameters of Flours

The data of the colorimetric indices considered two different methodologies of flour production. Table 3 showed the samples obtained according to methods A and B.

As can be seen, the values of the three colorimetric parameters, as expected, increased with greater flour integration. The data concerning the flours produced according to the method B showed that the trend of the flours does not change with respect to the previous one, albeit with a significant difference in the colors of the flours.

Comparing the data from the two methods, higher values were highlighted in flours produced with method B than with method A, a more evident result in pure flours, which is reflected both in the flour and in the bread color.

Samples produced according to method A tended to retain a color closer to the fresh product, especially for the bracts, which have a green color.

Method B tended to oxidize the product first, consequently eliminating the typical green color of the fresh product and making the flour very similar to a wholemeal one [47], which could be more easily accepted by the consumer.

The statistical factorial analysis confirms a significant difference in the flours re-garding the percentage variation in the different samples (Supplementary Table S4). In the case of the method used in relation to the samples, it would seem to influence the variation of the brown index less if compared to those of yellow and red index (Supplementary Table S5). Finally, the various flour integrations in relation to the two different methods show a high statistical variability (Supplementary Table S6).

#### 3.1.3. Cluster Analysis (Hierarchical Cluster Analysis and K-Means Cluster Analysis) in Flours

The whole dataset relating to the measured variables for the flours obtained with drying methods A and B, including both pure flours and mixed flours produced with different parts of artichoke with different percentage of additions, was utilized after data pretreatment for hierarchical cluster analysis, with distance measurement based on the squared Euclidean distance and the single-linkage (nearest neighbor) method of clustering.

**Figure 1:**
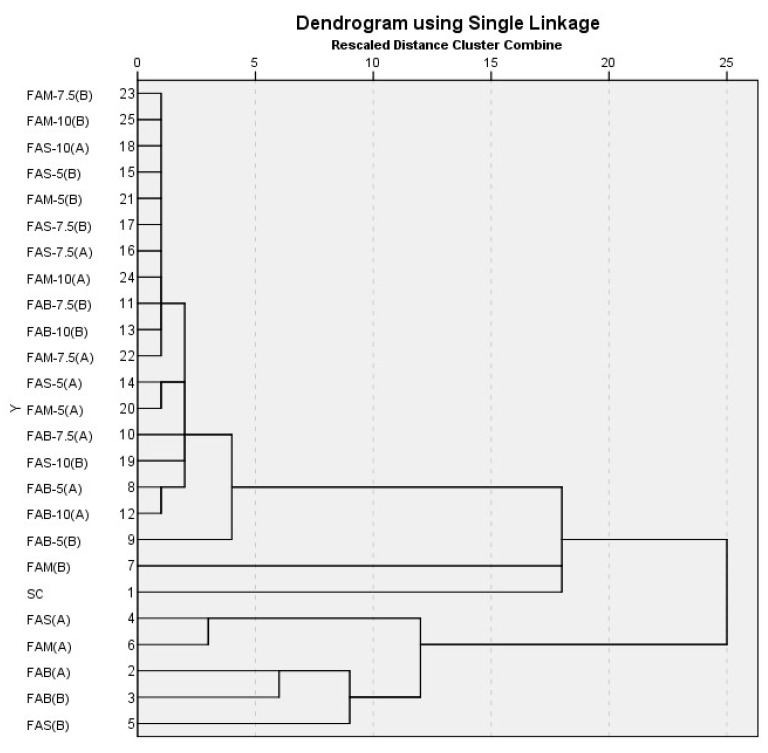
Dendrogram of hierarchical cluster analysis in flours. SC = semolina control 100%; FAB = flour of artichoke bracts to the 5/7.5/10/100%; FAS-5% = flour of artichoke stems to the 5/7.5/10/100%; FAM-5% = mixes 5/7.5/10/100%.

In Figure 1, we present the dendrogram resulting from hierarchical cluster analysis. A five-cluster solution identified three clusters, further segmented, and two single branches: SC and FAM (B). A first cluster included most of the mixed flours, apart from the pair, FAS (A) and FAM (A), joined in a cluster, and the triplet, FAB (A), FAB (B), and FAS (B), which form its own cluster.

The five-cluster solution was the conclusion of the hierarchical cluster analysis that was input to the K-means cluster analysis algorithm. SC formed a cluster of its own (cluster 1), like FAM (B) (cluster 3). The second cluster included the pair, FAB (A) and FAB (B); the fourth cluster comprised FAS (A), FAS (B), and FAM (A); the fifth was a large cluster including all the mixed flours.

As regards the distance to the cluster center, the smaller the value, the closer it is to the middle of that cluster, i.e., the centroid, and the more representative that flour (pure or mixed) is of that cluster. Conversely, the flour (pure or mixed) with the higher value is the least representative of that cluster. From our results, SC and FAM (B) were the centroids of their own group, of which they were the only components; the distances of the two components of cluster 2, (FAB method A and method B), were identical; FAS (A) was the centroid of cluster 4; FAM-10 (A) was the centroid of cluster 5.

**Figure 2:**
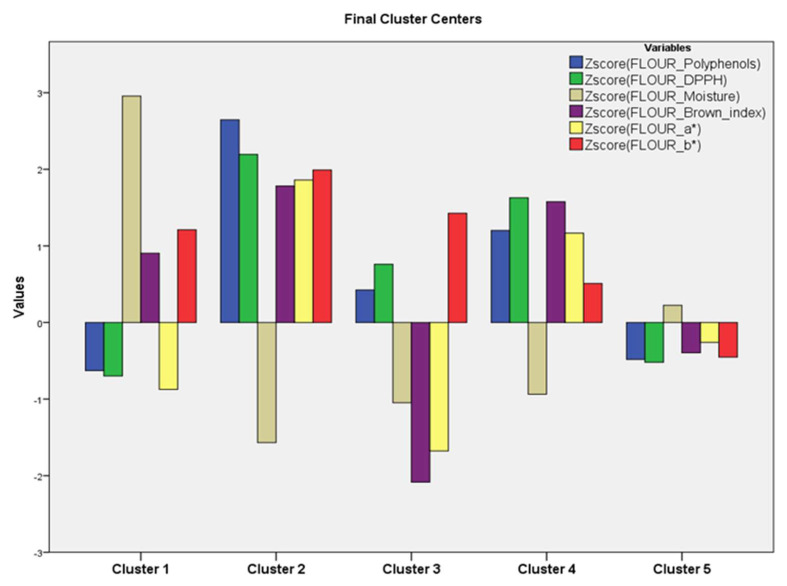
K-means cluster analysis in flours: quali- and quantitative fingerprint of each cluster, based on the scores of each variable. SC = semolina control 100%; FAB = flour of artichoke bracts to the 5/7.5/10/100%; FAS-5% = flour of artichoke stems to the 5/7.5/10/100%; FAM-5% = mixes 5/7.5/10/100%.

The graphical representation of the final cluster centers, based on the scores of each variable, which are specific to each cluster, gives us the quali-quantitative footprint of each of them (Figure 2). Cluster 1, represented solely by SC, showed the highest moisture values; cluster 2, FAB (A) and (B), stood out as having the highest values for most variables except for moisture, of which it showed the lowest values. The lowest FLOUR_Brown_index and FLOUR_a* values belonged to cluster 3 flours. Members of cluster 4 showed average values for most of the variables, while members of cluster 5 exhibited the lowest mean values.

Based on the data provided in the ANOVA table, the significance level was not applicable for testing the hypothesis regarding the mean variables. This is because the dispersion analysis results are solely descriptive, as the groups were intentionally formed based on the distances between them in the multidimensional space. Nevertheless, we can gain valuable insights into the significance of different mean variables in cluster formation by carefully analyzing the differences between the F-ratios. It is possible to observe that the variables DPPH and polyphenols, showing the highest value of F, had the maximum influence in forming the clusters. On the other hand, variables FLOUR_a* and FLOUR_b* had the least influence.

The clear distinction between the pure artichoke flours (clusters 2, 3, and 4) and sem-olina flour (cluster 1) was confirmed. The clustering of the pure flours of the various portions of artichoke was influenced by the two different methods.

The pure artichoke flours exhibited physiological characteristics which proportionally affected the integrated flours at various percentages of integration. Consequently, the integrated flours (cluster 5) possessed their own characteristics due to this interaction, distinguishable from the pure artichoke flours.

### 3.2. Breads

#### 3.2.1. Technological Analysis of Breads

Table 4 shows the data on the physicochemical characteristics of the breads produced according to the indicated methodology over the 4 days of storage in order to assess whether the different artichoke flours had influenced the parameters analyzed.

At T_0_, the moisture content of the samples fortified with the various artichoke flours increased compared to the control (33.10 g/100 g) and, specifically, the samples fortified with artichoke stem flour (39.66 g/100 g) in FAS 7.5% (B) showed a higher content than the two remaining types.

Both with bract flour and stem flour, a reduction in volume was observed as the integration percentages increased, with the highest values recorded in the control bread (361.00 cm^3^) and lowest in FAB-10% (A) (245.00 cm^3^). Regarding the height of the breads, a specular behavior in the volume was observed, with maximum values in the control bread (7.65 cm) and minimum values in FAB-7.5% (A) (4.90 cm). The weight was also affected by the moisture content of the breads, showing higher values in the integrated flours than in the control (132.76 g). There was a statistical difference between the breads integrated with bract flour compared to the other two types, which maintain similar values. The increase in weight of the supplemented breads compared to the control was also supported by the presence of fibers, as found by Fendri et al. [48] on fibrous matrices other than the artichoke. The bread had medium-high porosity in al-most all sample, except for the control and FAS-5% (A) (5.00).

As far as the moisture content of the samples is concerned, it was observed to decrease from 33.10 g/100 g (T_0_) to 29.45 g/100 g (T_4_) in the control, compared to the other samples supplemented with flours of the different artichoke portions, which showed a lower reduction in moisture due to the higher presence of fiber, which exerts a greater water retention action, maintaining the moisture content at T_4_ above 32 g/100 g. The rate of dehydration decreased over time due to interaction forces related to the different matrices, and it is also influenced by the degree of porosity and pore size [49]. The moisture content of the loaves, considering the size range of 100–250 g, complied with the provisions of law 580/1967 [50], which set the parameters at 31 g/100 g with the addition of D.P.R. 502/1998 [51], which gives a tolerance of 10% more in the case of the addition of flours other than wheat.

Concerning the other parameters, such as volume, height, and weight, a gradual reduction was noted from T_0_ to T_4_. In the control bread, there was a sharper reduction in these parameters, compared to those supplemented with the various artichoke flours, which had similar reductions over the four days.

The progressive reduction in volume, as reported in the literature, was typical for breads with increasing amounts of fibers [52,53], as well as for weight and height [54].

Finally, the porosity results showed a physiological reduction in pores from T_0_ to T_4_, as also found by other authors, especially in correlation with the grain size of the added flours [55] during the storage, causing a decrease in porosity and suggesting a denser and more compact texture compared to the control.

The factorial statistics, considering the sample and percentage as parameters (Supplementary Tables S7–S12) at the three different survey times (T_0_, T_2_, T_4_), demonstrated no statistical differences related to the use of flours produced with different methods; rather, they confirmed the percentage of the flour and the different samples (FAB, FAS, FAM) used as the discriminating parameters.

The results obtained, albeit with a decrease in volume, demonstrated an interesting technological potential that artichoke flours impart to breads, improving both the structural parameters and the bread yield thanks to the high water absorption values of the dough.

#### 3.2.2. TPA (Texture Profile Analysis) of the Breads Supplemented with Different Percentages of Additions

The texture of bread is extremely important in defining its quality and consumer acceptability, as it tends to vary with storage over time [56,57].

Texture profile analysis (TPA) revealed the influence of artichoke flour integration on the bread structure. Hardness is a specific mechanical property of a solid food, and it can be defined as the maximum force (N) required to achieve a given deformation [58,59]. In our tests, the hardness value was the result obtained from the first compression cycle (Table 5) of TPA. The increase in hardness is the most visible indication of the staling process during the storage of bread [60]. Table 5 shows how this parameter increases as the percentage of added artichoke flour rises, in agreement with the results found by Frutos et al. [59] in breads obtained by the integration of artichoke fibers, also confirming what was observed by other authors [61,62]. 

In particular, it was observed that FAB-10% (A) showed the highest significant (*p* ≤ 0.001) hardness values at the three-time intervals assessed, compared to all other samples. On the contrary, breads enriched with stems flour were softer than bracts breads, showing higher values at T4 in FAS-10%. The hardness in the mixed flour breads showed results more similar to stems breads, with no significant statistical differences.

Regarding the staling process of bread, the flours from the various parts of the artichoke were meliorative, giving a more stable structure over time, albeit with a greater firmness with respect to control. Among the three different flours added, it seems that the stems flour conferred a greater resistance to the staling process, characterized by its capacity to retain its softness during the considered storage time.

Concerning the control sample, a reduction in crust texture was observed between the time interval T_2_ and T_4_, caused by the internal moisture variation, which presumably brought above a rebalancing of the texture, as also indicated by Crowley et al. [63].

Springiness is a parameter used to define the freshness of the product and results in a more or less brittle crumb [64,65]. It is a measure of elasticity [66] and it is defined as the rate at which a deformed sample returns to its original size and shape [67]. In Table 5, it is possible to observe that at T_0_, the theses, supplemented with different percentages of artichoke flour, showed higher values compared to the control bread within a range of 0.91–1.00, albeit with a slight decrease from 5% to 10% supplementation, but with no significant statistical differences. This trend changed during storage, and on the second and fourth days of storage, the springiness of the breads integrated with artichoke flour was lower than the control bread, implying that the breads were less elastic and more compact than the semolina bread. Thus, springiness may be inversely correlated to cohesiveness, as more elastic doughs are less cohesive. This decrease in springiness has also been found by other authors for fiber-enriched breads [61,68].

Cohesiveness represents the extent to which the sample could be deformed before breaking [69], indicating the strength of the internal bonds in the sample [67]. It is related to the moisture content and the strength of the network surrounding the crumb cells [70], and in fact, low values of cohesiveness result in a bread with a crumb that crumbles easily [64,71]. From Table 5, it can be noted that at T_0_, cohesiveness in the samples with bract flour increased as the percentage of integration increased, while in the samples with stems and mixes, the values significantly decreased (*p* ≤ 0.001) from 5 to 10% integration. Similar trends were observed during storage, with a more pronounced decrease on the fourth day of storage, in which the bread with artichoke flour showed lower values than the control bread, with significant statistical differences with respect to the other samples. This latter finding might suggest that the use of artichoke flour in the formulation of semolina bread could favor the development of a crumb with a thicker and firmer structure [72].

Gumminess and chewiness also reported statistically significant differences, with the exception of gumminess at T_4_, for which no significant differences emerged from the statistical processing. In particular, at T_0_, breads with artichoke flour recorded significantly higher values than semolina-only bread, with values tending to increase as the percentage of integration increased. Among the various theses, at T_0_, breads with bract flour showed the highest values. This positive correlation is also observed during the storage bread, in which the values increase significantly compared to T_0_, desptie remaining below the gumminess and chewiness values recorded by the control at T_2_ and T_4_. Furthermore, the positive trend in chewiness was in line with that observed by Frutos et al. [59] in breads obtained with the addition of artichoke fibers.

Therefore, hardness and chewiness increases were in accordance with the results obtained by other authors [70,73] in the formulation of bread with artichoke flour.

Statistical factorial analysis at T_0_ (Supplementary Tables S13–S15), T_2_ (Supplementary Tables S16–S18), and T_4_ (Supplementary Tables S19–S21) showed significant differences for all TPA parameters influenced by the method, i.e., the percentage of integrated flour and the type of flour. This analysis highlights the different technological aspects conferred first by the flours produced from the various artichoke parts and by their different percentages of use, as already found by Canale et al. [7], and second by the method, albeit to a lesser extent.

#### 3.2.3. Polyphenols Content, Antioxidant Activity, and Water Activity in Breads with Different Integration Percentages

Table 6 shows polyphenols content, antioxidant activity, and water activity in breads with different integration percentages.

As regards polyphenols, it is possible to observe a significant lowering of the values compared to the polyphenols content found in the flours, ranging from 0.01 mg GAE/g in the control bread to 0.57 mg GAE/g in FAB-10% (method A).

The polyphenols in the FAB-10% sample exhibited higher values compared to the other thesis; moreover, comparing the two methods, it is noted that the samples obtained via method A had higher polyphenols content than those obviated via method B, except for integrations with the flour of artichoke stems, which showed a higher content (Supplementary Tables S22–S24).

Furthermore, as apparent from Table 6, the antioxidant activity of the samples was strongly correlated with their phenolic content, suggesting that polyphenols are the main component responsible for the antioxidant properties in the experiments.

The decrease in the quantity of polyphenols and of the antioxidant activity in the artichoke is caused by the high temperatures and the type of cooking method [74], and it is also found for matrices other than artichoke, such as chickpeas [75] and grape seed flours [76], unlike other studies involving the addition of tomato, beetroot, and carrot juices, which showed an increase in polyphenol content and antioxidant activity in the bread [77].

The water activity values showed a range from 0.63 in FAM-5% (B) to 0.90 in FAB-5% (B). No statistical difference was found between the breads produced using the two methods, only a decrease in concentrations from 5% to 10% was observed in the case of bract flours, and an increase from 5% to 10% in stems flours. In the case of breads produced with the mix of flours, the trend was similar to that of the bract’s flours, but less clear-cut.

#### 3.2.4. Color of Bread

Table 7 shows the color data of the samples obtained according to methods A and B.

The results highlight that the colorimetric indices of the crust tend to increase with the higher integration of artichoke flour, with a darker coloring in the theses produced according to method A. The highest crust brown index values were recorded in the samples with 10% supplementation for bract flours (56.93, method B), stem flours (61.35, method A), and mixes (57.85, method A). The red index was highest in the control (16.93) and lowest in FAB-10% (B) (6.97), while the yellow index ranged from 28.55 in FAB-5%(B) to 19.83 in FAS-10% (A).

In the crumb, we observed the same trend as for the crust, albeit with method B giving a darker brown index, with a range from 55.67 in FAB-100% (B) to 25.88 in the control bread, a red index between 4.71 in FAB-10% (A) and −2.20 in the control, and a yellow index between 24.11 (control) and 19.15 (FAS-10%, B).

#### 3.2.5. Cluster Analysis (Hierarchical Cluster Analysis and K-Means Cluster Analysis) in Breads

The dataset of the quali- and quantitative parameters detected in breads produced with flours (pure and mixed), prepared as described in Table 1, was utilized after data pretreatment for hierarchical cluster analysis adopting the ward linkage method of clustering.

**Figure 3:**
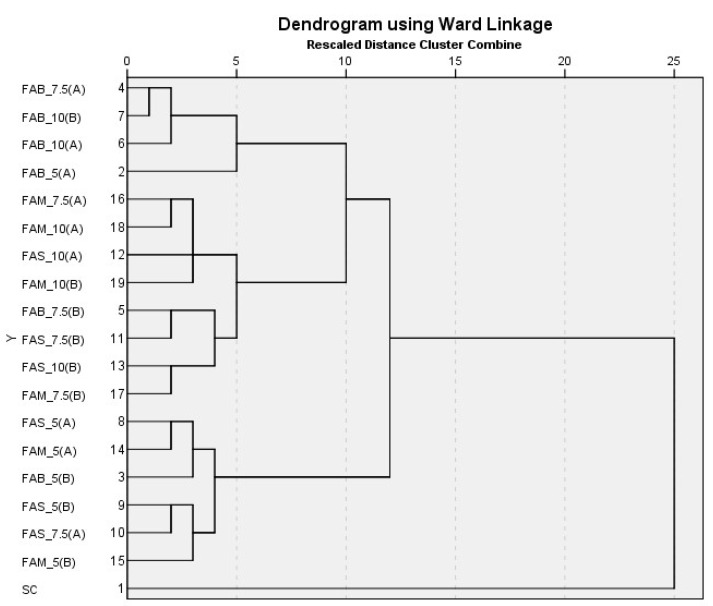
Dendrogram of hierarchical cluster analysis in breads. SC = bread with semolina control 100%; FAB = bread of artichoke bracts to the 5/7.5/10/100%; FAS-5% = bread of artichoke stems to the 5/7.5/10/100%; FAM-5% = bread of mixes 5/7.5/10/100%.

In Figure 3, we present the dendrogram resulting from hierarchical cluster analysis. A four-cluster solution identified three clusters, further segmented, and one single branch with SC as a unique component.

The four-cluster solution of the hierarchical cluster analysis on breads was used as input for the K-means cluster analysis algorithm. Moreover, in this case, SC formed a cluster of its own (cluster 1). The breads obtained with the flour mixtures were instead distributed in the other three clusters, where FAM_7.5 (B), FAS_5 (B), and FAB_7.5 (A) represented the centroids of clusters 2, 3, and 4, respectively.

**Figure 4:**
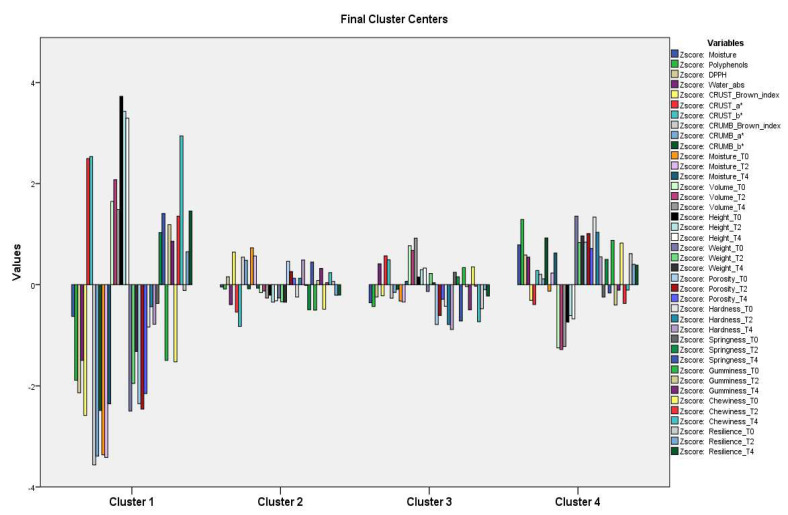
K-means cluster analysis in breads: quali-quantitative fingerprint of each cluster, based on the scores of each variable. SC = bread with semolina control 100%; FAB = bread of artichoke bracts to the 5/7.5/10/100%; FAS-5% = bread of artichoke stems to the 5/7.5/10/100%; FAM-5% = bread of mixes 5/7.5/10/100%.

Figure 4 shows the quali- and quantitative footprint of each cluster, obtained via the scores of each variable. SC, a unique component of cluster 1, was distinguished by higher values of Height_T0, Height_T2, Height_T4, Chewiness_T4, CRUST_b*, CRUST_a*, and Volume_T2 in particular, and for the lower values of CRUMB_Brown index, CRUMB_a*, Moisture_T0, and Moisture_T2. Clusters 2 and 3 showed the lowest mean values, while the values of cluster 4 were intermediate between cluster 1 and those of clusters 2 and 3.

Looking at the ANOVA results, in particular at Fisher’s F, the variables that most influenced the segmentation were Height_T0 and T2, Moisture_T0 and T2, and CRUMB_Brown index. The ones that had the least impact were Springiness_T0, Resilience_T2 and T0, Gumminess_T2, and Chewiness_T2.

The SC bread had its own set of characteristics (cluster 1): at higher integration percentages (7.5% and 10%), FAM and FAS tended to cluster together (cluster 2), as did FAB (cluster 4). At the 5% percentage, the breads of different origins formed a single cluster (cluster 3), with no differences among them being highlighted.

## 4. Conclusions

Owing to the importance of the reuse of artichoke processing waste and its relatively low economic impact, combined with a potential functional effect conferred by the artichoke, artichoke waste is one of the agrifood waste substances with great potential in the production of bakery products.

The analysis of the polyphenols and of the antioxidant activity, used as discriminants between the two different drying methods adopted, highlights a marked difference when speaking of flours.

The quantity of polyphenols and the antioxidant activity of the flours of the various artichoke parts showed a clear difference between pure flours (which have values in line with other studies carried out on similar flours) and integrated flours. The integration at 5, 7.5, and 10% proportionally increased the quantity of polyphenols, making the 10% bract flours produced with method A better than all the others. The data obtained showed that method B allowed for the production darker flours, which give a color very similar to wholemeal breads. The flours produced with method B were therefore more browned, and present a greater opportunity in the production of bakery products by eliminating the typical greenish color of method A, which may not be well accepted by the consumer.

In the case of breads, cooking processes tend to be destructive for most polyphenols by evening out the initial differences of the raw material between oxidized and non-oxidized products. Cooking has almost halved the polyphenol content and the antioxidant activity in the various breads integrated with the flours of the various parts of the artichoke.

The addition of artichoke flour has, however, conferred on the breads a higher content of polyphenols and a greater antioxidant activity, albeit in quantities that will not be of influence in terms of the health benefits related to their consumption.

This aspect should draw attention to other aspects related to the use of artichoke flour, such as the important technological characteristics capable of producing breads. The breads combined with artichoke flour, compared to the control, did not suffer significant weight losses, maintaining an almost constant moisture content during the four days of the test.

This aspect is very interesting as, although the supplemented bread has a lower porosity and volume than the 100% semolina control, the ability to hydrate and maintain freshness for several days is of considerable importance both to its use as a fresh product and as an industrial short-shelf-life product.

Therefore, the flours and breads produced with method B offer raw materials and food products in line with the required market standards.

## Figures and Tables

**Table 1:** Ingredients for the production of breads with different integration percentages.

Bread Type	Re-Milled Semolina (g)	Bracts/ Stem/Mix Flour (g)	Yeast Solution (mL)	Salt/Sugar Solution (mL)	Ascorbic Acid Solution (mL)	Distilled Water (mL)
SC (tester)	200	0	30	30	20	43.5
FAB-5% (A)	190	10	30	30	20	50.1
FAB-5% (B)	190	10	30	30	20	50.1
FAB-7.5% (A)	185	15	30	30	20	50.1
FAB-7.5% (B)	185	15	30	30	20	50.1
FAB-10% (A)	180	20	30	30	20	49.9
FAB-10% (B)	180	20	30	30	20	49.9
FAS-5% (A)	190	10	30	30	20	52.1
FAS-5% (B)	190	10	30	30	20	52.1
FAS-7.5% (A)	185	15	30	30	20	57.6
FAS-7.5% (B)	185	15	30	30	20	57.6
FAS-10% (A)	180	20	30	30	20	60.2
FAS-10% (B)	180	20	30	30	20	60.2
FAM-5% (A)	190	10	30	30	20	52.7
FAM-5% (B)	190	10	30	30	20	52.7
FAM-7.5% (A)	185	15	30	30	20	57.1
FAM-7.5% (B)	185	15	30	30	20	57.1
FAM-10% (A)	180	20	30	30	20	58.1
FAM-10% (B)	180	20	30	30	20	58.1

SC = bread with semolina control 100%; FAB = bread of artichoke bracts to the 5/7.5/10/100%; FAS-5% = bread of artichoke stems to the 5/7.5/10/100%; FAM-5% = bread of mixes 5/7.5/10/100%.

**Table 2:** Polyphenols content and antioxidant activity in flours produced according to method A and B, at different flour concentrations of bracts, stems and stem–bracts mix (0, 5, 7.5, 10%) as well as 100% bracts, stems and mix.

Sample	Polyphenols (mg GAE/g d.m.)	DPPH (mg Trolox eq/g d.m.)
*Pure flours*		
^1^SC	0.05 ± 0.00 h	0.05 ± 0.00 d
^2^FAB (A)	9.09 ± 0.12 a	8.59 ± 0.012 a
FAB (B)	6.98 ± 0.04 b	6.51 ± 0.13 ab
^3^FAS (A)	3.40 ± 0.18 d	6.18 ± 0.25 a–c
FAS (B)	4.93 ± 0.11 c	5.40 ± 0.97 bc
^4^FAM (A)	5.21 ± 0.13 c	6.66 ± 1.92 ab
FAM (B)	2.62 ± 0.36 e	3.83 ± 0.09 c
*Mixes*		
FAB-5% (A)	0.50 ± 0.01 f–h	0.47 ± 0.01 d
FAB-5% (B)	0.40 ± 0.00 gh	0.37 ± 0.01 d
FAB-7.5% (A)	0.73 ± 0.01 fg	0.69 ± 0.01 d
FAB-7.5% (B)	0.57 ± 0.00 f–h	0.53 ± 0.01 d
FAB-10% (A)	0.96 ± 0.02 f	0.90 ± 0.02 d
FAB-10% (B)	0.75 ± 0.00 fg	0.69 ± 0.01 d
FAS-5% (A)	0.22 ± 0.01 gh	0.42 ± 0.07 d
FAS-5% (B)	0.30 ± 0.00 gh	0.31 ± 0.04 d
FAS-7.5% (A)	0.13 ± 0.01 h	0.51 ± 0.02 d
FAS-7.5% (B)	0.17 ± 0.01 h	0.45 ± 0.07 d
FAS-10% (A)	0.39 ± 0.02 gh	0.66 ± 0.03 d
FAS-10% (B)	0.54 ± 0.01 fgh	0.58 ± 0.09 d
FAM-5% (A)	0.31 ± 0.00 gh	0.38 ± 0.10 d
FAM-5% (B)	0.18 ± 0.02 h	0.23 ± 0.00 d
FAM-7.5% (A)	0.18 ± 0.02 h	0.54 ± 0.15 d
FAM-7.5% (B)	0.11 ± 0.01 h	0.33 ± 0.00 d
FAM-10% (A)	0.57 ± 0.01 f–h	0.71 ± 0.20 d
FAM-10% (B)	0.31 ± 0.04 gh	0.42 ± 0.01 d

Data are expressed as mean ± standard deviation. Values in a column indicated by different letters are significantly different (*p* ≤ 0.001) based on Tukey’s HSD. ^1^SC = semolina control 100%; ^2^FAB = flour of artichoke bracts to the 5/7.5/10/100%; ^3^FAS-5% = flour of artichoke stems to the 5/7.5/10/100%; ^4^FAM-5% = mixes 5/7.5/10/100%.

**Table 3:** Colorimetric parameters of the samples of flours produced according to method A and B at different flour concentrations of bracts, stems, and stems–bracts mix (0, 5, 7.5, 10%), as well as 100% bracts, stems, and mix.

Sample	Brown Index (100-L)	a*	b*
*Pure flours*			
^1^SC	10.26 ± 0.01 l	−2.38 ± 0.00 q	17.17 ± 0.00 ab
^2^FAB (A)	34.56 ± 0.01 c	−1.21 ± 0.01 p	16.94 ± 0.01 a–c
FAB (B)	38.70 ± 0.01 b	2.91 ± 0.00 a	18.02 ± 0.00 a
^3^FAS (A)	44.69 ± 0.01 a	2.68 ± 0.02 b	17.53 ± 0.01 a
FAS (B)	41.36 ± 0.00 b	2.29 ± 0.01 c	15.74 ± 0.01 d–f
^4^FAM (A)	39.31 ± 0.04 b	0.71 ± 0.02 f	17.55 ± 0.01 a
FAM (B)	39.40 ± 0.03 b	2.36 ± 0.01 c	15.28 ± 0.01 d–g
*Mixes*			
FAB-5% (A)	22.75 ± 0.01 h–k	−2.01 ± 0.01 p	15.27 ± 0.01 d–g
FAB-5% (B)	17.75 ± 0.03 i	−0.42 ± 0.01 k	15.98 ± 0.01 c–e
FAB-7.5% (A)	22.72 ± 0.02 h–k	−1.93 ± 0.01 op	15.55 ± 0.02 d–f
FAB-7.5% (B)	25.74 ± 0.12 f–h	0.83 ± 0.01 e	15.97 ± 0.04 c–e
FAB-10% (A)	23.84 ± 0.01 g–j	−1.87 ± 0.01 o	15.68 ± 0.01 d–f
FAB-10% (B)	26.31 ± 0.01 d–f	1.02 ± 0.01 d	16.21 ± 0.02 b–d
FAS-5% (A)	20.71 ± 0.01 ki	−0.44 ± 0.03 k	15.09 ± 0.04 d–g
FAS-5% (B)	23.37 ± 0.04 g–k	0.08 ± 0.01 h	14.58 ± 0.01 f–h
FAS-7.5% (A)	24.15 ± 0.69 g–j	−0.11 ± 0.01 j	14.71 ± 0.01 f–h
FAS-7.5% (B)	25.63 ± 0.07 f–h	0.36 ± 0.00 g	14.31 ± 0.01 g–h
FAS-10% (A)	27.55 ± 0.02 de	0.34 ± 0.02 g	14.93 ± 0.04 e–h
FAS-10% (B)	28.81 ± 2.52 d	0.66 ± 0.08 f	13.75 ± 1.02 h
FAM-5% (A)	20.76 ± 0.01 ki	−1.12 ± 0.01 m	15.09 ± 0.01 d–g
FAM-5% (B)	21.72 ± 0.01 jk	−0.14 ± 0.01 j	14.89 ± 0.01 e–h
FAM-7.5% (A)	23.59 ± 0.01 g–k	−1.01 ± 0.01 m	15.35 ± 0.01 d–g
FAM-7.5% (B)	24.32 ± 0.15 g–j	0.38 ± 0.00 g	15.14 ± 0.01 d–g
FAM-10% (A)	26.20 ± 0.01 d–f	−0.78 ± 0.01 l	15.08 ± 0.01 d–g
FAM-10% (B)	26.01 ± 0.00 d–f	0.65 ± 0.01 f	15.28 ± 0.01 d–g

Data are expressed as mean ± standard deviation. Values in a column indicated by different letters are significantly different (*p* ≤ 0.001) based on Tukey’s HSD. ^1^SC = semolina control 100%; ^2^FAB = flour of artichoke bracts to the 5/7.5/10/100%; ^3^FAS-5% = flour of artichoke stems to the 5/7.5/10/100%; ^4^FAM-5% = mixes 5/7.5/10/100%.

**Table 4:** Physical characteristics of the loaves at different percentages of integration with artichoke flour and the staling process from T_0_ to T_4_.

Time	Sample	Moisture (g/100 g)	Volume (cm^3^)	Height (cm)	Weight (g)	Porosity *
	SC	33.10 ± 0.19 d	361.00 ± 5.66 a	7.65 ± 0.21 a	132.76 ± 0.54 b	5.00 ± 0.00 c
T_0_	FAB-5% (A)	37.79 ± 0.19 abc	300.00 ± 0.00 c–f	5.15 ± 0.21 bcd	136.89 ± 0.83 ab	6.00 ± 0.00 abc
	FAB-5% (B)	36.48 ± 0.54 c	335.00 ± 7.07 a–d	5.50 ± 0.00 bcd	136.94 ± 0.04 ab	5.75 ± 0.35 abc
	FAB-7.5% (A)	37.75 ± 0.04 abc	262.50 ± 3.54 fg	4.90 ± 0.00 d	137.82 ± 0.42 a	7.00 ± 0.00 a
	FAB-7.5% (B)	37.57 ± 0.25 abc	297.50 ± 3.54 c–g	5.28 ± 0.18 bcd	137.96 ± 0.57 a	7.00 ± 0.00 a
	FAB-10% (A)	37.81 ± 0.83 abc	245.00 ± 7.07 g	4.95 ± 0.21 cd	137.85 ± 1.07 a	6.75 ± 0.35 ab
	FAB-10% (B)	37.77 ± 0.18 abc	267.50 ± 17.68 fg	5.10 ± 0.14 bcd	137.73 ± 0.27 a	6.75 ± 0.00 ab
	FAS-5% (A)	36.92 ± 0.39 bc	350.00 ± 28.28 abc	5.68 ± 0.11 bcd	135.09 ± 0.26 ab	5.00 ± 0.00 c
	FAS-5% (B)	37.69 ± 0.28 abc	327.50 ± 3.54 a–e	5.65 ± 0.07 bcd	135.45 ± 1.38 ab	6.00 ± 0.35 abc
	FAS-7.5% (A)	38.18 ± 0.49 abc	355.00 ± 7.07 ab	5.70 ± 0.144 bc	134.40 ± 0.58 ab	6.25 ± 0.00 abc
	FAS-7.5% (B)	39.66 ± 0.23 a	325.00 ± 0.00 a–e	5.43 ± 0.11 bcd	136.13 ± 1.81 ab	6.75 ± 0.35 ab
	FAS-10% (A)	38.72 ± 0.50 ab	325.00 ± 7.07 a–e	5.43 ± 0.11 bcd	135.30 ± 0.33 ab	6.75 ± 0.71 ab
	FAS-10% (B)	39.10 ± 0.37 ab	302.50 ± 3.54 b–f	5.33 ± 0.04 bcd	135.95 ± 0.26 ab	6.25 ± 0.35 abc
	FAM-5% (A)	37.62 ± 0.01 abc	330.00 ± 7.07 a–e	5.78 ± 0.18 b	136.49 ± 0.46 ab	6.00 ± 0.00 abc
	FAM-5% (B)	38.15 ± 0.22 abc	304.00 ± 5.66 b–f	5.54 ± 0.08 bcd	135.77 ± 0.86 ab	7.00 ± 0.00 a
	FAM-7.5% (A)	39.21 ± 0.42 a	302.50 ± 3.54 b–f	5.32 ± 0.16 bcd	135.41 ± 0.12 ab	6.75 ± 0.00 ab
	FAM-7.5% (B)	39.10 ± 0.08 ab	306.50 ± 2.12 b–f	5.51 ± 0.14 bcd	135.74 ± 0.27 ab	7.00 ± 0.35 a
	FAM-10% (A)	38.47 ± 0.53 abc	277.50 ± 3.54 efg	5.12 ± 0.02 bcd	135.24 ± 1.12 ab	6.50 ± 0.00 ab
	FAM-10% (B)	39.18 ± 0.20 a	282.50 ± 3.54 d–g	5.27 ± 0.06 bcd	136.15 ± 0.04 ab	7.00 ± 0.71 a
T_2_	SC	32.67 ± 0.58 b	348.75 ± 8.84 a	7.20 ± 0.28 a	124.10 ± 0.39 i	5.50 ± 0.00 c
	FAB-5% (A)	36.09 ± 1.69 ab	277.50 ± 3.54 b–f	4.90 ± 0.21 bcd	132.85 ± 0.40 a–d	6.25 ± 0.00 abc
	FAB-5% (B)	36.54 ± 2.07 ab	311.25 ± 1.77 a–d	5.40 ± 0.14 bcd	134.39 ± 0.15 ab	6.25 ± 0.00 abc
	FAB-7.5% (A)	38.52 ± 0.50 a	257.50 ± 3.54 def	4.88 ± 0.04 bcd	133.29 ± 0.38 abc	7.25 ± 0.35 a
	FAB-7.5% (B)	38.14 ± 0.06 a	258.00 ± 9.90 def	5.10 ± 0.14 bcd	129.27 ± 1.07 d–g	7.25 ± 0.00 a
	FAB-10% (A)	38.00 ± 0.34 a	240.00 ± 7.07 f	4.83 ± 0.07 bcd	131.22 ± 0.93 b–f	7.00 ± 0.35 ab
	FAB-10% (B)	37.03 ± 0.44 ab	245.00 ± 21.21 ef	4.87 ± 0.05 bcd	135.38 ± 0.14 a	7.00 ± 0.00 ab
	FAS-5% (A)	37.10 ± 0.22 ab	330.00 ± 28.28 ab	5.60 ± 0.07 b	129.39 ± 0.85 d–g	6.00 ± 0.00 bc
	FAS-5% (B)	37.46 ± 0.00 a	298.00 ± 2.83 a–e	5.55 ± 0.07 bc	129.11 ± 0.03 efg	6.00 ± 0.00 bc
	FAS-7.5% (A)	37.60 ± 1.60 a	319.50 ± 7.78 abc	5.53 ± 0.32 bc	125.35 ± 0.16 hi	6.25 ± 0.00 abc
	FAS-7.5% (B)	37.43 ± 1.06 a	302.50 ± 3.54 a–d	5.35 ± 0.14 bcd	128.61 ± 0.25 e–h	6.25 ± 0.35 abc
	FAS-10% (A)	38.22 ± 1.60 a	302.50 ± 3.54 a–d	5.38 ± 0.04 bcd	125.77 ± 0.17 ghi	6.75 ± 0.71 abc
	FAS-10% (B)	38.71 ± 1.63 a	272.50 ± 3.54 c–f	5.20 ± 0.14 bcd	127.91 ± 0.12 fgh	6.75 ± 0.71 abc
	FAM-5% (A)	37.77 ± 0.59 a	310.00 ± 2.83 a–d	5.61 ± 0.06 b	131.82 ± 0.11 a–e	6.75 ± 0.00 abc
	FAM-5% (B)	37.68 ± 0.21 a	277.50 ± 3.54 b–f	5.20 ± 0.14 bcd	129.63 ± 0.43 def	6.00 ± 0.00 bc
	FAM-7.5% (A)	39.19 ± 0.46 a	292.50 ± 3.54 a–f	5.18 ± 0.11 bcd	130.67 ± 0.16 c–f	7.00 ± 0.00 ab
	FAM-7.5% (B)	38.57 ± 0.70 a	294.00 ± 1.41 a–f	5.00 ± 0.00 bcd	128.40 ± 0.01 e–h	7.00 ± 0.71 ab
	FAM-10% (A)	38.91 ± 0.19 a	269.00 ± 1.41 c–f	4.70 ± 0.14 cd	129.98 ± 1.82 c–f	7.00 ± 0.00 ab
	FAM-10% (B)	38.58 ± 0.26 a	262.50 ± 3.54 def	4.55 ± 0.14 d	129.68 ± 0.14 c–f	7.00 ± 0.00 ab
T_4_	SC	29.45 ± 0.73 c	316.50 ± 4.95 ab	6.86 ± 0.23 a	118.81 ± 0.11 g	6.00 ± 0.00 a
	FAB-5% (A)	35.69 ± 0.78 ab	272.50 ± 3.54 a–e	4.78 ± 0.04 bcd	127.31 ± 0.42 b	7.00 ± 0.00 a
	FAB-5% (B)	37.38 ± 0.16 a	301.00 ± 1.41 abc	5.34 ± 0.20 bc	131.20 ± 0.03 a	7.00 ± 0.35 a
	FAB-7.5% (A)	36.65 ± 0.93 ab	252.50 ± 3.54 cde	4.65 ± 0.10 cd	130.28 ± 0.23 a	7.50 ± 0.71 a
	FAB-7.5% (B)	35.37 ± 0.59 abc	254.00 ± 8.49 cde	4.95 ± 0.21 bcd	127.15 ± 0.55 b	7.50 ± 0.00 a
	FAB-10% (A)	34.77 ± 1.97 abc	235.00 ± 7.07 e	4.67 ± 0.05 cd	127.10 ± 0.45 b	7.63 ± 0.35 a
	FAB-10% (B)	36.66 ± 0.71 ab	241.00 ± 19.80 de	4.86 ± 0.06 bcd	131.45 ± 0.32 a	7.25 ± 0.00 a
	FAS-5% (A)	32.33 ± 1.25 abc	326.50 ± 30.41 a	5.54 ± 0.08 b	126.85 ± 0.76 b	6.75 ± 0.35 a
	FAS-5% (B)	36.08 ± 0.00 ab	295.00 ± 2.83 a–d	5.39 ± 0.01 bc	127.53 ± 0.25 b	6.75 ± 0.35 a
	FAS-7.5% (A)	32.71 ± 1.58 abc	316.50 ± 6.36 ab	5.40 ± 0.14 bc	122.19 ± 0.26 ef	7.50 ± 0.35 a
	FAS-7.5% (B)	34.59 ± 2.45 abc	252.50 ± 3.54 cde	5.31 ± 0.13 bcd	122.19 ± 0.08 ef	7.00 ± 0.35 a
	FAS-10% (A)	30.74 ± 0.13 bc	282.50 ± 3.54 a–e	5.23 ± 0.11 bcd	118.83 ± 0.05 g	7.50 ± 1.41 a
	FAS-10% (B)	36.52 ± 0.36 ab	272.50 ± 3.54 a–e	5.10 ± 0.14 bcd	124.21 ± 0.25 d	7.50 ± 0.35 a
	FAM-5% (A)	33.74 ± 1.41 abc	299.00 ± 1.41 abc	5.40 ± 0.14 bc	127.40 ± 0.23 b	7.25 ± 0.35 a
	FAM-5% (B)	35.33 ± 1.26 abc	278.50 ± 2.12 a–e	5.03 ± 0.11 bcd	126.26 ± 0.04 bc	6.25 ± 0.35 a
	FAM-7.5% (A)	32.41 ± 2.76 abc	276.25 ± 1.77 a–e	5.14 ± 0.08 bcd	123.72 ± 0.37 de	7.25 ± 0.35 a
	FAM-7.5% (B)	33.94 ± 0.28 abc	281.00 ± 1.41 a–e	4.88 ± 0.18 bcd	121.11 ± 0.09 f	7.25 ± 0.71 a
	FAM-10% (A)	36.25 ± 1.59 ab	260.00 ± 2.83 cde	4.59 ± 0.16 cd	124.28 ± 0.05 d	7.25 ± 0.35 a
	FAM-10% (B)	35.11 ± 0.73 abc	262.50 ± 3.54 b–e	4.48 ± 0.22 d	124.55 ± 0.45 cd	7.75 ± 0.35 a

* Scale 1–8; 1 = non-uniform structure: large and irregular cells; 8 = uniform compact structure: small and regular cells. Data are expressed as mean ± standard deviation. Values in a column at each storage time, indicated by different letters are significantly different (T_0_ *p* ≤ 0.001; T_2_ *p* ≤ 0.001; moisture *p* ≤ 0.01; T_4_: volume, height, weight: *p* ≤ 0.001; moisture and porosity: *p* ≤ 0.01), based on Tukey’s HSD. SC = bread with semolina control 100%; FAB = bread of artichoke bracts to the 5/7.5/10/100%; FAS-5% = Bread of artichoke stems to the 5/7.5/10/100%; FAM-5% = bread of mixes 5/7.5/10/100%.

**Table 5:** Texture profile analysis data in the four days of storage.

Time	Sample	Hardness (N)	Springiness	Cohesiveness	Gumminess (N)	Chewiness (N × mm)
	SC-100%	6.44 ± 0.93 e–h	0.95 ± 0.07 ab	0.71 ± 0.02 bcd	6.50 ± 0.68 h	5.64 ± 0.34 m
T_0_	FAB-5% (A)	14.15 ± 0.35 abc	1.00 ± 0.01 a	0.56 ± 0.01 e	24.68 ± 0.47 cd	24.29 ± 0.03 e
	FAB-5% (B)	9.01 ± 0.53 c–g	0.96 ± 0.01 ab	0.77 ± 0.06 abc	44.83 ± 0.85 a	43.81 ± 0.02 a
	FAB-7.5% (A)	15.25 ± 0.07 ab	0.99 ± 0.01 ab	0.70 ± 0.01 bcd	44.91 ± 0.16 a	44.07 ± 0.08 a
	FAB-7.5% (B)	11.75 ± 0.92 b–e	0.94 ± 0.01 ab	0.81 ± 0.01 ab	22.80 ± 0.04 d	21.45 ± 0.13 f
	FAB-10% (A)	19.35 ± 2.47 a	0.96 ± 0.00 ab	0.82 ± 0.02 ab	30.63 ± 0.02 bc	29.23 ± 0.08 d
	FAB-10% (B)	13.55 ± 0.64 bcd	0.94 ± 0.01 ab	0.70 ± 0.02 bcd	45.92 ± 0.40 a	42.95 ± 0.07 a
	FAS-5% (A)	3.42 ± 0.27 h	0.96 ± 0.01 ab	0.63 ± 0.01 de	42.47 ± 0.74 a	41.25 ± 0.02 b
	FAS-5% (B)	10.27 ± 0.47 b–f	1.00 ± 0.01 a	0.72 ± 0.01 a–d	19.52 ± 0.39 def	19.59 ± 0.12 gh
	FAS-7.5% (A)	7.80 ± 0.37 e–h	1.00 ± 0.01 a	0.66 ± 0.01 cde	30.48 ± 0.42 bc	30.30 ± 0.44 d
	FAS-7.5% (B)	8.38 ± 0.87 d–h	0.98 ± 0.01 ab	0.83 ± 0.01 a	14.11 ± 0.12 fg	13.74 ± 0.03 ij
	FAS-10% (A)	9.21 ± 1.02 c–f	0.96 ± 0.00 ab	0.68 ± 0.01 cde	11.80 ± 0.28 gh	11.22 ± 0.07 k
	FAS-10% (B)	9.10 ± 0.37 c–g	0.94 ± 0.01 ab	0.72 ± 0.01 a–d	33.43 ± 4.41 b	34.16 ± 0.10 c
	FAM-5% (A)	3.71 ± 0.82 gh	0.91 ± 0.02 b	0.76 ± 0.03 abc	14.17 ± 0.16 fg	12.61 ± 0.11 j
	FAM-5% (B)	8.01 ± 0.73 e–h	0.97 ± 0.01 ab	0.67 ± 0.01 cde	10.10 ± 0.18 gh	9.80 ± 0.31 l
	FAM-7.5% (A)	11.30 ± 1.56 b–f	1.00 ± 0.01 a	0.70 ± 0.01 bcd	20.80 ± 0.18 de	20.72 ± 0.22 fg
	FAM-7.5% (B)	10.25 ± 0.35 b–f	0.95 ± 0.01 ab	0.72 ± 0.01 a–d	15.77 ± 0.11 efg	14.90 ± 0.04 i
	FAM-10% (A)	7.61 ± 0.60 e–h	0.91 ± 0.01 b	0.67 ± 0.03 cde	20.11 ± 0.05 def	18.65 ± 0.49 h
	FAM-10% (B)	6.31 ± 0.01 fgh	0.99 ± 0.01 ab	0.75 ± 0.01 a–d	24.53 ± 0.03 cd	23.97 ± 0.01 e
T_2_	SC-100%	20.40 ± 0.57 c–f	0.99 ± 0.01 ab	0.81 ± 0.04 ab	64.68 ± 0.62 b	62.93 ± 0.24 abc
	FAB-5% (A)	16.85 ± 0.07 def	1.00 ± 0.00 a	0.90 ± 0.05 a	26.34 ± 0.62 e–h	26.29 ± 0.55 fgh
	FAB-5% (B)	13.05 ± 0.21 f	0.93 ± 0.01 ab	0.63 ± 0.08 b	51.04 ± 0.23 c	47.06 ± 0.20 de
	FAB-7.5% (A)	22.85 ± 1.34 c–f	0.97 ± 0.01 ab	0.79 ± 0.05 ab	29.57 ± 0.80 ef	29.49 ± 0.56 fgh
	FAB-7.5% (B)	37.15 ± 4.31 ab	0.97 ± 0.01 ab	0.80 ± 0.05 ab	29.75 ± 1.07 ef	29.82 ± 0.41 fgh
	FAB-10% (A)	47.80 ± 0.00 a	0.95 ± 0.01 ab	0.79 ± 0.03 ab	41.08 ± 0.66 cd	39.57 ± 0.68 ef
	FAB-10% (B)	24.90 ± 2.26 b–f	0.98 ± 0.02 ab	0.78 ± 0.01 ab	18.34 ± 5.25 g–j	17.80 ± 4.77 ghi
	FAS-5% (A)	18.50 ± 3.96 c–f	0.94 ± 0.01 ab	0.77 ± 0.04 ab	9.01 ± 0.92 j	8.40 ± 0.88 i
	FAS-5% (B)	15.15 ± 1.63 ef	0.94 ± 0.01 ab	0.70 ± 0.03 ab	49.13 ± 0.23 c	46.93 ± 0.18 de
	FAS-7.5% (A)	23.25 ± 1.63 b–f	0.98 ± 0.03 ab	0.72 ± 0.04 ab	62.84 ± 0.23 b	59.50 ± 0.74 bcd
	FAS-7.5% (B)	27.15 ± 0.21 b–f	0.94 ± 0.08 ab	0.64 ± 0.01 ab	34.76 ± 0.35 de	34.88 ± 0.55 ef
	FAS-10% (A)	30.30 ± 0.57 bcd	0.85 ± 0.08 ab	0.80 ± 0.01 ab	12.06 ± 0.60 ij	11.23 ± 0.05 i
	FAS-10% (B)	31.85 ± 2.90 bc	0.81 ± 0.06 b	0.61 ± 0.10 b	92.81 ± 4.33 a	75.44 ± 8.97 a
	FAM-5% (A)	17.25 ± 1.63 def	0.90 ± 0.10 ab	0.78 ± 0.06 ab	28.06 ± 0.35 efg	26.95 ± 0.12 fgh
	FAM-5% (B)	27.10 ± 0.14 b–f	0.88 ± 0.09 ab	0.74 ± 0.04 ab	19.77 ± 0.63 f–i	15.93 ± 0.62 hi
	FAM-7.5% (A)	15.55 ± 0.07 ef	0.96 ± 0.00 ab	0.87 ± 0.02 ab	49.89 ± 0.48 c	48.68 ± 0.80 cde
	FAM-7.5% (B)	29.25 ± 3.04 b–e	0.97 ± 0.01 ab	0.70 ± 0.03 ab	66.45 ± 0.81 b	64.77 ± 0.47 ab
	FAM-10% (A)	16.55 ± 2.62 def	0.95 ± 0.03 ab	0.79 ± 0.02 ab	30.72 ± 0.43 e	30.73 ± 0.78 fg
	FAM-10% (B)	24.50 ± 5.52 b–f	0.97 ± 0.04 ab	0.80 ± 0.02 ab	16.36 ± 0.65 hij	15.91 ± 0.13 hi
T_4_	SC-100%	23.35 ± 2.33 c–f	0.95 ± 0.01 a	0.81 ± 0.05 a	277.90 ± 0.95	263.70 ± 2.73 a
	FAB-5% (A)	28.50 ± 1.56 b–f	0.74 ± 0.02 c	0.42 ± 0.05 c	60.96 ± 2.45	43.15 ± 5.64 c
	FAB-5% (B)	18.95 ± 1.06 ef	0.92 ± 0.01 ab	0.74 ± 0.02 ab	103.35 ± 1.37	80.80 ± 23.30 bc
	FAB-7.5% (A)	26.70 ± 3.11 c–f	0.84 ± 0.01 abc	0.71 ± 0.01 abc	82.64 ± 5.20	65.76 ± 9.39 bc
	FAB-7.5% (B)	41.60 ± 0.00 abc	0.87 ± 0.02 abc	0.74 ± 0.02 ab	172.41 ± 10.15	130.55 ± 39.48 abc
	FAB-10% (A)	50.45 ± 1.63 a	0.88 ± 0.03 abc	0.72 ± 0.01 abc	118.97 ± 8.63	104.46 ± 11.63 bc
	FAB-10% (B)	31.90 ± 2.40 a–f	0.87 ± 0.04 abc	0.69 ± 0.01 abc	149.76 ± 4.86	74.18 ± 87.27 bc
	FAS-5% (A)	16.95 ± 0.78 f	0.78 ± 0.03 bc	0.72 ± 0.01 abc	42.91 ± 3.26	30.82 ± 2.59 c
	FAS-5% (B)	23.70 ± 3.82 c–f	0.87 ± 0.01 abc	0.72 ± 0.00 abc	81.69 ± 1.29	71.02 ± 2.65 bc
	FAS-7.5% (A)	28.85 ± 2.19 b–f	0.84 ± 0.02 abc	0.70 ± 0.01 abc	62.63 ± 4.81	53.74 ± 3.42 c
	FAS-7.5% (B)	46.65 ± 5.59 ab	0.86 ± 0.01 abc	0.68 ± 0.04 abc	100.94 ± 3.85	82.70 ± 9.31 bc
	FAS-10% (A)	36.95 ± 1.06 a–e	0.86 ± 0.03 abc	0.57 ± 0.04 cd	100.25 ± 5.52	90.00 ± 2.04 bc
	FAS-10% (B)	28.55 ± 7.28 b–f	0.95 ± 0.02 a	0.74 ± 0.01 ab	127.29 ± 35.09	146.91 ± 0.45 abc
	FAM-5% (A)	16.40 ± 0.99 f	0.86 ± 0.01 abc	0.69 ± 0.01 abc	80.98 ± 3.42	73.62 ± 2.39 bc
	FAM-5% (B)	40.90 ± 2.83 abc	0.91 ± 0.01 ab	0.72 ± 0.00 abc	71.38 ± 0.15	66.82 ± 3.75 bc
	FAM-7.5% (A)	21.25 ± 0.35 def	0.87 ± 0.04 abc	0.64 ± 0.02 bc	103.33 ± 0.40	90.87 ± 0.93 bc
	FAM-7.5% (B)	48.95 ± 5.59 a	0.93 ± 0.07 ab	0.74 ± 0.05 ab	214.21 ± 16.08	200.54 ± 30.52 ab
	FAM-10% (A)	31.95 ± 3.04 a–f	0.86 ± 0.00 abc	0.59 ± 0.01 bc	125.59 ± 1.81	111.11 ± 2.06 bc
	FAM-10% (B)	40.60 ± 4.10 a–d	0.93 ± 0.01 ab	0.65 ± 0.02 bc	131.65 ± 1.51	114.18 ± 8.07 bc

Data are expressed as mean ± standard deviation. Values in a column at each storage time, indicated by different letters are significantly different (*p* ≤ 0.001), based on Tukey’s HSD; absence of letters indicate absence of significant differences. SC = bread with semolina control 100%; FAB = bread of artichoke bracts to the 5/7.5/10/100%; FAS-5% = bread of artichoke stems to the 5/7.5/10/100%; FAM-5% = Bread of mixes 5/7.5/10/100%.

**Table 6:** Polyphenols content, antioxidant activity, and water activity in breads with different integration percentages.

Sample	Polyphenols (mg GAE/g d.m.)	DPPH (mg Trolox eq/g d.m.)	Aw
SC	0.01 ± 0.01 i	n.d. *	0.64 ± 0.01 f
FAB-5% (A)	0.29 ± 0.01 d	0.32 ± 0.00 c–f	0.86 ± 0.03 ab
FAB-5% (B)	0.22 ± 0.01 ef	0.24 ± 0.03 fgh	0.90 ± 0.02 a
FAB-7.5% (A)	0.35 ± 0.00 b	0.44 ± 0.00 abc	0.87 ± 0.01 ab
FAB-7.5% (B)	0.29 ± 0.00 d	0.23 ± 0.05 fgh	0.86 ± 0.01 ab
FAB-10% (A)	0.57 ± 0.01 a	0.55 ± 0.00 a	0.77 ± 0.02 b–e
FAB-10% (B)	0.37 ± 0.00 b	0.42 ± 0.00 a–d	0.78 ± 0.03 bcd
FAS-5% (A)	0.14 ± 0.00 h	0.27 ± 0.01 e–h	0.78 ± 0.02 abc
FAS-5% (B)	0.14 ± 0.00 h	0.17 ± 0.03 gh	0.74 ± 0.01 c–f
FAS-7.5% (A)	0.18 ± 0.00 gh	0.41 ± 0.00 bcd	0.76 ± 0.05 b–e
FAS-7.5% (B)	0.24 ± 0.01 ef	0.28 ± 0.00 efg	0.86 ± 0.02 ab
FAS-10% (A)	0.23 ± 0.00 ef	0.54 ± 0.00 a	0.85 ± 0.03 ab
FAS-10% (B)	0.30 ± 0.02 cd	0.45 ± 0.04 abc	0.82 ± 0.01 abc
FAM-5% (A)	0.17 ± 0.00 gh	0.30 ± 0.04 d–g	0.78 ± 0.02 bcd
FAM-5% (B)	0.13 ± 0.01 h	0.14 ± 0.02 h	0.63 ± 0.02 f
FAM-7.5% (A)	0.26 ± 0.00 de	0.39 ± 0.02 cde	0.66 ± 0.01 ef
FAM-7.5% (B)	0.15 ± 0.01 gh	0.17 ± 0.01 gh	0.67 ± 0.01 def
FAM-10% (A)	0.34 ± 0.01 bc	0.54 ± 0.00 ab	0.65 ± 0.01 f
FAM-10% (B)	0.20 ± 0.01 fg	0.25 ± 0.01 fgh	0.73 ± 0.02 c–f

* Not detectable. Data are expressed as mean ± standard deviation. Values in a column indicated by different letters are significantly different (*p* ≤ 0.001) based on Tukey’s HSD. SC = bread with semolina control 100%; FAB = bread of artichoke bracts to the 5/7.5/10/100%; FAS-5% = bread of artichoke stems to the 5/7.5/10/100%; FAM-5% = bread of mixes 5/7.5/10/100%.

**Table 7:** Colorimetric parameters of the crust and crumb of loaves at different concentrations of bracts, stems flour, and of the stems-bracts mix for methods A and B (data presented as mean ± standard deviation).

Sample	Crust			Crumb		
	Brown Index (100-L)	a*	b*	Brown Index (100-L)	a*	b*
SC	58.56 ± 3.03 abcd	16.38 ± 0.33 a	22.43 ± 0.81 bc	25.88 ± 0.01 l	−2.20 ± 0.03 h	24.11 ± 0.06 a
FAB-5% (A)	48.09 ± 3.25 d	8.29 ± 0.10 bc	28.30 ± 1.14 a	42.95 ± 0.39 i	1.27 ± 0.28 e	20.41 ± 0.06 bcd
FAB-5% (B)	49.59 ± 0.89 cd	9.19 ± 0.16 bc	28.55 ± 1.03 ab	49.48 ± 0.12 defghi	2.78 ± 0.04 bcdefg	20.15 ± 0.04 bcdefg
FAB-7.5% (A)	48.37 ± 2.78 d	8.45 ± 0.65 bc	27.91 ± 0.47 ab	47.20 ± 0.35 fghi	2.02 ± 0.04 ef	19.46 ± 0.45 defgh
FAB-7.5% (B)	53.30 ± 3.13 abcd	8.94 ± 0.35 bc	24.50 ± 2.84 abc	51.45 ± 0.78 cdef	3.92 ± 0.11 abc	20.46 ± 0.78 bcd
FAB-10% (A)	56.28 ± 2.40 abcd	8.03 ± 1.34 bc	23.47 ± 1.16 abc	55.07 ± 0.12 abcd	4.71 ± 0.11 a	20.32 ± 0.31 bcd
FAB-10% (B) FAS-5% (A)	56.93 ± 1.67 abcd 51.89 ± 1.94 bcd	6.97 ± 0.74 c 8.55 ± 5.10 bc	21.93 ± 0.08 bc 24.88 ± 2.28 abc	55.67 ± 0.30 abc 48.51 ± 0.54 efghi	2.46 ± 0.28 def 2.47 ± 0.40 def	18.91 ± 0.16 gh 19.73 ± 0.20 cdefgh
FAS-5% (A) FAS-5% (B)	51.89 ± 1.94 bcd 51.90 ± 1.39 bcd	8.55 ± 5.10 bc 9.84 ± 0.25 bc	24.88 ± 2.28 abc 25.56 ± 0.74 abc	48.51 ± 0.54 efghi 46.54 ± 0.45 ghi	2.47 ± 0.40 def 2.50 ± 0.06 def	19.73 ± 0.20 cdefgh 20.14 ± 0.14 bcdef
FAS-7.5% (A) FAS-7.5% (B)	57.50 ± 0.21 abc 59.93 ± 0.54 ab	11.37 ± 1.39 abc 12.07 ± 0.49	22.20 ± 2.97 bc 21.93 ± 1.90 bc	51.28 ± 0.18 cdefg 50.43 ± 0.13 defgh	3.47 ± 0.39 abcd 3.51 ± 0.15 abcd	20.62 ± 0.48 bc 19.59 ± 0.02 defgh
FAS-10% (A) FAS-10% (B)	61.35 ± 1.64 a 54.60 ± 5.25 abcd	10.14 ± 0.62 abc 8.66 ± 0.30 bc	19.83 ± 3.59 c 23.33 ± 1.33 abc	58.49 ± 0.06 a 54.61 ± 0.06 abcd	3.95 ± 0.01 abc 3.79 ± 0.04 abc	18.83 ± 0.08 g 19.15 ± 0.05 fgh
FAM-5% (A) FAM-5% (B)	54.21 ± 2.33 abcd 52.98 ± 2.74 abcd	11.22 ± 0.11 bc 12.49 ± 0.92 abc	26.02 ± 0.23 ab 27.12 ± 0.85 ab	46.20 ± 0.07 hi 46.76 ± 0.29 fghi	2.00 ± 0.02 ef 2.70 ± 0.11 cdef	21.00 ± 0.03 b 20.45 ± 0.13 bcd
FAM-7.5% (A) FAM-7.5% (B)	54.03 ± 0.98 abcd 56.70 ± 0.03 abcd	10.60 ± 0.03 ab 11.29 ± 0.22 bc	25.60 ± 0.07 abc 22.93 ± 0.08 bc	52.13 ± 0.08 bcde 54.68 ± 0.13 abcd	2.78 ± 0.14 cdef 2.15 ± 0.04 ef	20.17 ± 0.09 bcde 19.86 ± 0.03 cdefg
FAM-10% (A) FAM-10% (B)	57.85 ± 0.33 abc 55.83 ± 1.05 abcd	10.07 ± 0.47 abc 10.27 ± 0.66 bc	22.21 ± 0.89 bc 24.70 ± 0.88 abc	56.63 ± 0.11 ab 54.17 ± 0.06 abcd	3.14 ± 0.05 bcde 4.07 ± 0.06 ab	19.30 ± 0.03 efgh 20.04 ± 0.02 bcdef

Data are expressed as mean ± standard deviation. Values in a column indicated by different letters are significantly different (*p* ≤ 0.001) based on Tukey’s HSD. SC = bread with semolina control 100%; FAB = bread of artichoke bracts to the 5/7.5/10/100%; FAS-5% = bread of artichoke stems to the 5/7.5/10/100%; FAM-5% = bread of mixes 5/7.5/10/100%.

## Data Availability

All available data are reported in the paper.

## Supplementary Materials

The following supporting information can be downloaded at: https://www.mdpi.com/article/10.3390/foods12183419/s1, Table S1: Polyphenols content and antioxidant activity, in pure flours and their integrations at different percentages. Factorial ANOVA for “sample-method”; Table S2: Polyphenols content and antioxidant activity, in pure flours and their integrations at different percentages. Factorial ANOVA for “sample-percentages”; Table S3: Polyphenols content and antioxidant activity, in pure flours and their integrations at different percentages. Factorial ANOVA for “Percentages-Method”; Table S4: Colorimetric parameters of the samples of flour produced according to method A and B, at different flour concentrations of bracts, stems and stem-bracts mix (0, 5, 7.5, 10%) as well as 100% bracts, stems and mix. Factorial ANOVA for “sample-percentages”; Table S5: Colorimetric parameters of the samples of flour produced according to method A and B, at different flour concentrations of bracts, stems and stem-bracts mix (0, 5, 7.5, 10%) as well as 100% bracts, stems and mix. Factorial ANOVA for “sample-method”; Table S6: Colorimetric parameters of the samples of flour produced according to method A and B, at different flour concentrations of bracts, stems and stem-bracts mix (0, 5, 7.5, 10%) as well as 100% bracts, stems and mix. Factorial ANOVA for “Percentages-Method”; Table S7: Physical characteristics of the loaves at different percentages of integration with artichoke flour and staling process from T0 to T4. Factorial ANOVA for “sample-method” a T0; Table S8: Physical characteristics of the loaves at different percentages of integration with artichoke flour and staling process from T0 to T4. Factorial ANOVA for “sample-method” a T2; Table S9: Physical characteristics of the loaves at different percentages of integration with artichoke flour and staling process from T0 to T4. Factorial ANOVA for “sample-method” a T4; Table S10: Physical characteristics of the loaves at different percentages of integration with artichoke flour and staling process from T0 to T4. Factorial ANOVA for “sample-percentages” a T0; Table S11: Physical characteristics of the loaves at different percentages of integration with artichoke flour and staling process from T0 to T4. Factorial ANOVA for “sample-percentages” a T2; Table S12: Physical characteristics of the loaves at different percentages of integration with artichoke flour and staling process from T0 to T4. Factorial ANOVA for “sample-percentages” a T4; Table S13: Texture Profile Analysis data in the five days of storage. Factorial ANOVA for “sample-percentages” a T0; Table S14: Texture Profile Analysis data in the five days of storage. Factorial ANOVA for “sample-method” a T0; Table S15: Texture Profile Analysis data in the five days of storage. Factorial ANOVA for “percentages-method” T0; Table S16: Texture Profile Analysis data in the five days of storage. Factorial ANOVA for “sample-percentages” a T2; Table S17: Texture Profile Analysis data in the five days of storage. Factorial ANOVA for “sample-method” a T2; Table S18: Texture Profile Analysis data in the five days of storage. Factorial ANOVA for “percentages-method” T2; Table S19: Texture Profile Analysis data in the five days of storage. Factorial ANOVA for “sample-percentages” a T4; Table S20: Texture Profile Analysis data in the five days of storage. Factorial ANOVA for “sample-method” a T4; Table S21: Texture Profile Analysis data in the five days of storage. Factorial ANOVA for “percentages-method” T4; Table S22: Factorial ANOVA for “Sampling-Percentage”; Table S23: Polyphenols content, antioxidant activity and water activity, in breads with different integration percentages. Factorial ANOVA for “Sampling-Method”; Table S24: Polyphenols content, antioxidant activity and water activity, in breads with different integration percentages. Factorial ANOVA for “Percentage-Sampling”.
