# Supplementary material for: Artichoke Industrial Waste in Durum Wheat Bread: Effects of Two Different Preparation and Drying Methods of Flours and Evaluation of Quality Parameters during Short Storage
Source: Foods. 2023 Sep 14;12(18):3419. doi: 10.3390/foods12183419 (PMC10527894; doi:10.3390/foods12183419)
Supplement: Supplementary file 1 [file foods-12-03419-s001.zip › foods-2589435-supplementary.pdf]

Supplementary Materials: factorial ANOVA

Table S1 - Polyphenols content and antioxidant activity, in pure flours and their integrations at different percentages. Factorial ANOVA for "sample-method"

Sample	Polyphenols (mgGAE/g d.m)	DPPH (mg trolox eq/g d.m)
Method A		
FAB	2.82±3.87a	2.66±0.07
FAS	1.04±2.97d	1.94±0.25
FAM	1.57±1.41c	2.07±0.27
Method B		
FAB	2.17±2.05b	2.02±0.84
FAS	1.48±2.17c	1.68±1.86
FAM	0.80±1.09e	1.20±2.96

Data are expressed as means ± standard deviations. Different letters indicate p≤0.001, no letter not significant.

Table S2 - Polyphenols content and antioxidant activity, in pure flours and their integrations at different percentages. Factorial ANOVA for "sample-percentages"

Sample	Polyphenols (mgGAE/g d.m)	DPPH (mg trolox eq/g d.m)
Pure flours		
FAB	8.03±1.22a	7.55±0.29a
FAS	4.16±0.89b	5.80±1.04b
FAM	3.92±1.51b	5.25±3.77b
Mixes		
FAB-5	0.45±0.06de	0.42±0.01c
FAB-7.5%	0.65±0.09cd	0.61±0.02c
FAB-10%	0.85±0.31c	0.80±0.03c
FAS-5%	0.26±0.04e	0.36±0.06c
FAS-7.5%	0.15±0.13e	0.48±0.08c
FAS-10%	0.46±0.09de	0.62±0.10c
FAM-5%	0.25±0.08e	0.31±0.19c
FAM-7.5%	0.15±0.04e	0.44±0.28c
FAM-10%	0.44±0.15de	0.57±0.38c

Data are expressed as means ± standard deviations. Different letters indicate p≤0.001, no letter not significant.

Table S3 - Polyphenols content and antioxidant activity, in pure flours and their integrations at different percentages. Factorial ANOVA for "Percentages-Method"

Sample	Polyphenols (mgGAE/g d.m)	DPPH (mg trolox eq/g d.m)
Method A		
5%	0.36±0.13d	0.42±0.11c
7%	0.35±0.29d	0.58±0.16c
10%	0.64±0.26c	0.76±0.21c
100%	5.90±2.60a	7.15±2.14a
Method B		
5%	0.29±0.10d	0.31±0.18c
7%	0.27±0.22d	0.44±0.26c

10%	0.53±0.20cd	0.57±0.35c
100%	4.84±1.96b	5.25±3.51b

Data are expressed as means ± standard deviations. Different letters indicate p≤0.001, no letter not significant.

Table S4 - Colorimetric parameters of the samples of flour produced according to method A and B, at different flour concentrations of bracts, stems and stem-bracts mix (0, 5, 7.5, 10%) as well as 100% bracts, stems and mix. Factorial ANOVA for "sample-percentages"

Sample	Brown index	a*	b*
FAB-5%	20.25±2.89e	-1.22±0.92h	15.62±0.41ab
FAB-7.5%	24.23±1.75c	-0.55±1.59g	15.76±0.25ab
FAB-10%	25.07±1.42bc	-0.42±1.67f	15.94±0.30a
FAS-5%	22.04±1.53d	-0.18±0.30d	14.83±0.30cde
FAS-7.5%	24.89±0.94bc	0.13±0.27b	14.51±0.23de
FAS-10%	28.18±1.63a	0.50±0.19a	14.34±0.90e
FAM-5%	21.24±0.55de	-0.63±0.56g	14.99±0.12cd
FAM-7.5%	23.95±0.43c	-0.31±0.80e	15.24±0.12bc
FAM-10%	26.10±0.11b	-0.07±0.82c	15.18±0.12bc

Data are expressed as means ± standard deviations. Different letters indicate (p≤0,05 Brown index, b*; p≤0,001 a*).

Table S5 - Colorimetric parameters of the samples of flour produced according to method A and B, at different flour concentrations of bracts, stems and stem-bracts mix (0, 5, 7.5, 10%) as well as 100% bracts, stems and mix. Factorial ANOVA for "sample-method"

Sample	Brown index	a*	b*
Method A			
FAB	23.10±0.57b	-1.94±0.07f	15.50±0.19ab
FAS	24.14±3.07b	-0.07±0.35d	14.91±0.17bc
FAM	23.51±2.43b	-0.97±0.16e	15.17±0.13b
Method B			
FAB	23.26±4.28b	0.48±0.70a	16.05±0.12a
FAS	25.93±2.69a	0.36±0.26b	14.21±0.59c

Data are expressed as means ± standard deviations. Different letters indicate (p≤0,05 Brown index; p≤0.001 a*, b*)

Table S6 - Colorimetric parameters of the samples of flour produced according to method A and B, at different flour concentrations of bracts, stems and stem-bracts mix (0, 5, 7.5, 10%) as well as 100% bracts, stems and mix. Factorial ANOVA for "Percentages-Method"

Sample	Brown index	a*	b*
Method A			
5%	21.41±1.04d	-1.19±0.70f	15.15±0.10
7%	23.48±0.72c	-1.01±0.82e	15.20±0.39
10%	25.86±1.68ab	-0.77±0.98d	15.23±0.36
Method B			
5%	20.95±2.58d	-0.16±0.22c	15.15±0.66
7%	25.23±0.71b	0.52±0.24b	15.14±0.74
10%	27.04±1.78a	0.77±0.19a	15.08±1.20

Data are expressed as means ± standard deviations. Different letters indicate (p≤0,01 Brown index; p≤0,001 a*), no letter not significant.

Table S7- Physical characteristics of the loaves at different percentages of integration with artichoke flour and staling process from T0 to T4. Factorial ANOVA for "sample-method" a T0

Campione	Moisture (g/100g)	Volume (cm^3)	Height (cm)	Weight (g)	Porosity (1-8)*

Method A					
FAB	37.78±0.38ab	269.17±25.38c	5.00±0.18c	137.52±0.80	6.58±0.49a
FAS	37.94±0.90ab	343.33±19.66a	5.60±0.16a	134,91±0.52	5.92±0.80b
FAM	38.44±0.77a	303.33±23.80b	5.40±0.32ab	135.71±0.81	6.75±0.42a
Method B					
FAB	37.27±0.68b	300.00±31.46b	5.29±0.21b	137.54±0.55	6.50±0.63a
FAS	38.82±0.94a	318.33±12.52ab	5.47±0.16ab	135.84±1.07	6.25±0.52ab
FAM	38.81±0.53a	297.67±12.21b	5.44±0.15ab	135.89±0.45	6.42±0.49ab

Data are expressed as means ± standard deviations. Different letters indicate ($p \leq 0.001$ moisture, volume; $p \leq 0.01$ height; $p \leq 0.05$ porosity). no letter not significant.

Table S8 - Physical characteristics of the loaves at different percentages of integration with artichoke flour and staling process from T0 to T4. Factorial ANOVA for "sample-method" a T2

Campione	Moisture (g/100g)	Volume (cm³)	Height (cm)	Weight (g)	Porosity (1-8)*
Method A					
FAB	37.54±1.40	258.33±17.22c	4.87±0.11b	132.45±1.09ab	6.83±0.52
FAS	37.64±1.14	317.33±18.13a	5.50±0.18a	126.84±2.03e	6.33±0.41
FAM	38.62±0.75	290.50±18.52ab	5.16±0.42ab	130.82±1.17bc	6.92±0.20
Method B					
FAB	37.24±1.20	271.42±33.11bc	5.12±0.26ab	133.01±2.97a	6.83±0.52
FAS	37.87±1.09	291.00±14.70ab	5.37±0.18a	128.54±0.56cd	6.33±0.41
FAM	38.28±0.58	278.00±14.28bc	4.92±0.31b	129.24±0.68cd	6.67±0.52

Data are expressed as means ± standard deviations. Different letters indicate $p \leq 0.001$, no letter not significant.

Table S9 - Physical characteristics of the loaves at different percentages of integration with artichoke flour and staling process from T0 to T4. Factorial ANOVA for "sample-method" a T4

Campione	Moisture (g/100g)	Volume (cm³)	Height (cm)	Weight (g)	Porosity (1-8)*
Method A					
FAB	35.70±1.03a	253.33±17.22b	4.70±0.08b	128.23±1.62b	7.38±0.31
FAS	31.93±1.48b	308.50±24.92a	5.39±0.17a	122.62±3.62e	7.25±0.42
FAM	34.14±1.85ab	278.42±17.60b	5.04±0.38ab	125.13±1.79c	7.25±0.27
Method B					
FAB	36.47±1.30a	265.33±29.84b	5.05±0.26ab	129.93±2.18a	7.25±0.27
FAS	35.73±1.28a	273.33±19.19b	5.27±0.16a	124.64±2.42cd	7.08±0.49
FAM	34.79±1.70a	274.00±9.19b	4.79±0.29b	123.97±2.35d	7.08±0.86

Data are expressed as means ± standard deviations. Different letters indicate ($p \leq 0.05$ moisture; $p \leq 0.001$ volume, height, weight). no letter not significant.

Table S10 - Physical characteristics of the loaves at different percentages of integration with artichoke flour and staling process from T0 to T4. Factorial ANOVA for "sample-percentages" a T0

Sample	Moisture (g/100g)	Volume (cm³)	Height (cm)	Weight (g)	Porosity (1-8)*
FAB-5%	37.13±0.83	317.50±20.62ab	5.33±0.24	136.92±0.48	5.88±0.25cd
FAB-7.5%	37.66±0.18	280.00±20.41cd	5.09±0.24	137.89±0.41	7.00±0.00a
FAB-10%	37.79±0.49	256.25±17.02d	5.03±0.17	137.79±0.64	6.75±0.29ab
FAS-5%	37.30±0.53	338.75±20.97a	5.66±0.08	135.27±0.84	5.38±0.48d
FAS-7.5%	38.92±0.91	340.00±17.80a	5.56±0.19	135.27±1.48	6.13±0.25bc

FAS-10%	38.91±0.42	313.75±13.77ab	5.38±0.09	135.60±0.47	6.75±0.29ab
FAM-5%	37.89±0.33	317.00±15.90ab	5.66±0.18	136.13±0.70	6.13±0.25bc
FAM-7.5%	39.16±0.26	304.50±3.32bc	5.42±0.16	135.57±0.26	6.88±0.25a
FAM-10%	38.83±0.53	280.00±4.08cd	5.19±0.10	135.70±0.83	6.75±0.50ab

Data are expressed as means ± standard deviations. Different letters indicate (p≤0.01 volume; p≤0.05 porosity), no letter not significant.

Table S11 - Physical characteristics of the loaves at different percentages of integration with artichoke flour and staling process from T0 to T4. Factorial ANOVA for "sample-percentages" a T2

Sample	Moisture (g/100g)	Volume (cm³)	Height (cm)	Weight (g)	Porosity (1-8)*
FAB-5%	36.32±1.57	294.38±19.62a	5.15±0.32b-e	133.62±0.92a	6.25±0.29bc
FAB-7.5%	38.33±0.36	257.75±6.08c	4.99±0.15de	131.28±2.41b	7.25±0.29a
FAB-10%	37.51±0.64	242.50±13.23c	4.85±0.05ef	133.30±2.46a	7.00±0.00a
FAS-5%	37.28±0.24	314.00±24.71a	5.58±0.06a	129.25±0.52c	6.00±0.00c
FAS-7.5%	37.51±1.11	311.00±10.98a	5.44±0.23ab	126.98±1.89d	6.25±0.29bc
FAS-10%	38.46±1.35	287.50±17.56ab	5.29±0.13a-d	126.84±1.24d	6.75±0.29ab
FAM-5%	37.72±0.37	293.75±18.95ab	5.41±0.25abc	130.72±1.29bc	6.38±0.48bc
FAM-7.5%	38.88±0.60	293.25±2.36ab	5.09±0.12cde	129.53±1.31bc	7.00±0.00a
FAM-10%	38.75±0.26	265.75±4.35bc	4.63±0.14f	129.83±1.07bc	7.00±0.00a

Data are expressed as means ± standard deviations. Different letters indicate (p≤0.05 height, porosity; p≤0.01 volume, weight), no letter not significant.

Table S12- Physical characteristics of the loaves at different percentages of integration with artichoke flour and staling process from T0 to T4. Factorial ANOVA for "sample-percentages" a T4

Sample	Moisture (g/100g)	Volume (cm³)	Height (cm)	Weight (g)	Porosity (1-8)*
FAB-5%	36.53±1.08	286.75±16.60	5.06±0.35bcd	129.26±2.26a	7.00±0.00
FAB-7.5%	36.01±0.97	253.25±5.38	4.80±0.22de	128.72±1.84a	7.50±0.00
FAB-10%	35.71±1.61	238.00±12.62	4.76±0.12de	129.27±2.53a	7.44±0.31
FAS-5%	34.21±2.32	310.75±25.33	5.47±0.10a	127.19±0.61b	6.75±0.29
FAS-7.5%	33.65±1.42	284.50±37.19	5.36±0.12ab	122.19±0.16d	7.25±0.29
FAS-10%	33.63±3.63	277.50±6.45	5.16±0.13abc	121.52±3.11d	7.50±0.41
FAM-5%	34.54±1.24	288.75±11.93	5.21±0.24abc	126.83±0.67b	6.75±0.65
FAM-7.5%	33.17±1.97	278.63±3.04	5.01±0.19cd	122.41±1.52d	7.25±0.65
FAM-10%	35.68±1.14	261.25±2.99	4.53±0.17e	124.41±0.31c	7.50±0.41

Data are expressed as means ± standard deviations. Different letters indicate (p≤0.05 height; p≤0.001 weight), no letter not significant.

Table S13 - Texture Profile Analysis data in the five days of storage. Factorial ANOVA for "sample-percentages" a T0

Sample	Hardness (N)	Springiness	Resilience	Gumminess (N)	Chewiness (N x mm)
FAB-5%	11.58±2.99bc	0.98±0.03ab	0.66±0.13c	34.76±11.65ab	34.05±11.27b
FAB-7.5%	13.50±2.09ab	0.96±0.03abc	0.75±0.06a	33.85±12.76b	32.76±13.06c
FAB-10%	16.45±3.66a	0.95±0.02bc	0.76±0.07a	38.27±8.83a	36.09±7.92a
FAS-5%	6.84±3.96e	0.98±0.02ab	0.67±0.05bc	30.99±13.26b	30.42±12.51d
FAS-7.5%	8.09±0.64de	0.99±0.01a	0.74±0.10ab	22.29±9.46cd	22.02±9.56ef
FAS-10%	9.15±0.63cde	0.95±0.01abc	0.70±0.03abc	22.61±12.75c	22.69±13.24e
FAM-5%	5.86±2.56e	0.94±0.04c	0.72±0.06abc	12.14±2.35e	11.21±1.63h

FAM-7.5%	10.78±1.10bcd	0.98±0.03ab	0.71±0.02abc	18.29±2.91d	17.81±3.36g
FAM-10%	6.96±0.82e	0.95±0.05bc	0.71±0.05abc	22.32±2.55cd	21.31±3.08f

Data are expressed as means ± standard deviations. Different letters indicate p⩽0.001, no letter not significant.

Table S14 - Texture Profile Analysis data in the five days of storage. Factorial ANOVA for “sample-method” a T0

Sample	Hardness (N)	Springiness	Resilience	Gumminess (N)	Chewiness (Nxmm)
Method A					
FAB	16.25±2.69a	0.98±0.02a	0.69±0.12b	33.40±9.30b	32.53±9.21b
FAS	6.81±2.75c	0.97±0.02a	0.65±0.02b	28.25±13.83c	27.59±13.59c
FAM	7.54±3.50c	0.94±0.05c	0.71±0.05ab	18.36±3.26e	17.33±3.78e
Method B					
FAB	11.44±2.12b	0.94±0.01bc	0.76±0.06a	37.85±11.67a	36.07±11.33a
FAS	9.25±0.98bc	0.97±0.03a	0.76±0.06a	22.35±9.13d	22.50±9.41d
FAM	8.19±1.80c	0.97±0.02ab	0.71±0.04ab	16.80±6.50e	16.22±6.42f

Data are expressed as means ± standard deviations. Different letters indicate p⩽0.001, no letter not significant.

Table S15 - Texture Profile Analysis data in the five days of storage. Factorial ANOVA for “percentages-method” T0

Sample	Hardness	Springiness	Resilience	Gumminess	Chewiness
Method A					
5%	7.09±5.48c	0.96±0.05bc	0.65±0.09c	27.11±12.80b	26.05±12.88c
7.50%	11.45±3.41ab	1.00±0.01a	0.69±0.03bc	32.06±10.85a	31.70±10.50b
10%	12.06±5.83a	0.94±0.03c	0.72±0.08b	20.84±8.44c	19.70±8.10e
Method B					
5%	9.09±1.11bc	0.97±0.02ab	0.72±0.05b	24.82±16.07b	24.40±15.66d
7.50%	10.13±1.62ab	0.95±0.02bc	0.79±0.05a	17.56±4.13d	16.70±3.72f
10%	9.65±3.28abc	0.95±0.03bc	0.72±0.03b	34.62±9.81a	33.69±8.50a

Data are expressed as means ± standard deviations. Different letters indicate p⩽0.001, no letter not significant.

Table S16 - Texture Profile Analysis data in the five days of storage. Factorial ANOVA for “sample-percentages” a T2

Sample	Hardness (N)	Springiness	Resilience	Gumminess (N)	Chewiness (N x mm)
FAB-5%	14.95±2.20d	0.96±0.04a	0.76±0.16	38.69±14.26c	36.68±12.00cd
FAB-7.5%	30.00±8.66ab	0.97±0.01a	0.79±0.04	29.66±0.78d	29.65±0.44de
FAB-10%	36.35±13.29a	0.96±0.02a	0.78±0.02	29.71±13.48d	28.68±12.88de
FAS-5%	16.83±3.14cd	0.94±0.01ab	0.74±0.05	29.07±23.17d	27.66±22.25de
FAS-7.5%	25.20±2.44bc	0.96±0.06a	0.68±0.06	48.80±16.21b	47.19±14.22b
FAS-10%	31.08±1.93ab	0.83±0.06b	0.70±0.12	52.43±46.69ab	43.33±37.43bc
FAM-5%	22.18±5.76bcd	0.89±0.08ab	0.76±0.05	23.91±4.80d	21.44±6.37e
FAM-7.5%	22.40±8.10bcd	0.97±0.01a	0.78±0.10	58.17±9.58a	56.72±9.31a
FAM-10%	20.53±5.79cd	0.96±0.03a	0.79±0.02	23.54±8.30d	23.32±8.57e

Data are expressed as means ± standard deviations. Different letters indicate p⩽0.001, no letter not significant.

Table S17 - Texture Profile Analysis data in the five days of storage. Factorial ANOVA for "sample-method" a T2

Sample	Hardness (N)	Springiness	Resilience	Gumminess (N)	Chewiness (N x mm)
Metodo A					
FAB	29.17±14.69a	0.97±0.02	0.82±0.07	32.33±6.95bc	31.78±6.22bc
FAS	24.02±5.65a	0.92±0.07	0.76±0.04	27.97±27.05c	26.37±25.70c
FAM	16.45±1.58b	0.94±0.05	0.81±0.05	36.22±10.66b	35.45±10.40b
Metodo B					
FAB	25.03±11.00a	0.96±0.03	0.73±0.09	33.04±15.04b	31.56±13.33bc
FAS	24.72±7.85a	0.90±0.08	0.65±0.06	58.90±27.11a	52.42±19.06a
FAM	26.95±3.53a	0.94±0.07	0.74±0.05	34.19±25.04b	32.20±25.23bc

Data are expressed as means ± standard deviations. Different letters indicate $p \leq 0.001$, no letter not significant.

Table S18 - Texture Profile Analysis data in the five days of storage. Factorial ANOVA for "percentages-method" T2

Sample	Hardness	Springiness	Resilience	Gumminess	Chewiness
Metodo A					
5%	17.53±2.06c	0.95±0.06	0.82±0.07	21.14±9.44d	20.54±9.43c
7.50%	20.55±3.99bc	0.97±0.02	0.79±0.07	47.43±15.01a	45.89±13.61a
10%	31.55±14.06a	0.92±0.07	0.79±0.02	27.95±13.16c	27.18±12.98c
Metodo B					
5%	18.43±6.82c	0.91±0.05	0.69±0.06	39.98±15.68b	36.64±16.05b
7.50%	31.18±5.27a	0.96±0.04	0.71±0.08	43.65±17.81ab	43.16±16.90ab
10%	27.08±4.74ab	0.92±0.09	0.73±0.10	42.50±39.09ab	36.38±30.61b

Data are expressed as means ± standard deviations. Different letters indicate $p \leq 0.001$, no letter not significant.

Table S19 - Texture Profile Analysis data in the five days of storage. Factorial ANOVA for "sample-percentages" a T4

Campione	Hardness (N)	Springiness	Resilience	Gumminess (N)	Chewiness (N x mm)
FAB-5%	23.73±5.62cd	0.83±0.10	0.58±0.19c	82.16±24.53cd	61.97±25.77bc
FAB-7.5%	34.15±8.79ab	0.85±0.02	0.72±0.02a	127.53±52.25ab	98.15±44.14abc
FAB-10%	41.18±10.84a	0.87±0.03	0.71±0.02ab	134.36±18.67ab	89.32±53.75abc
FAS-5%	20.33±4.50d	0.83±0.06	0.72±0.01a	62.30±22.48c	50.92±23.30c
FAS-7.5%	37.75±10.85ab	0.85±0.02	0.69±0.02ab	81.78±22.40cd	68.22±17.68bc
FAS-10%	32.75±6.45abc	0.90±0.05	0.65±0.10abc	113.77±25.78bc	118.46±32.88ab
FAM-5%	28.65±14.25bcd	0.88±0.03	0.70±0.02ab	76.18±5.89cd	70.22±4.69bc
FAM-7.5%	35.10±16.32ab	0.90±0.06	0.69±0.07ab	158.77±64.69a	145.71±65.73a
FAM-10%	36.28±5.80ab	0.89±0.04	0.62±0.04bc	128.62±3.75ab	112.64±5.12ab

Data are expressed as means ± standard deviations. Different letters indicate ($p \leq 0.001$ springiness, resilience, gumminess; $p \leq 0.01$ hardness; $p \leq 0.05$ chewiness), no letter not significant.

Table S20- Texture Profile Analysis data in the five days of storage. Factorial ANOVA for "sample-method" a T4

Campione	Hardness (N)	Springiness	Resilience	Gumminess (N)	Chewiness (N x mm)
Method A					
FAB	35.22±11.95ab	0.82±0.07	0.62±0.16d	87.52±26.62b	71.12±28.64
FAS	27.58±9.07bc	0.83±0.04	0.66±0.08bc	68.59±26.30c	58.19±26.77
FAM	23.20±7.26c	0.86±0.02	0.64±0.04cd	103.30±20.03b	91.87±16.85
Method B					
FAB	30.82±10.23bc	0.88±0.03	0.72±0.03a	141.84±31.89a	95.17±51.99
FAS	32.97±11.70b	0.89±0.04	0.71±0.03a	103.30±25.86b	100.21±36.81

FAM	43.48±5.40c	0.92±0.03	0.70±0.05ab	139.08±64.54a	127.18±62.29

Data are expressed as means ± standard deviations. Different letters indicate ($p \leq 0.001$ hardness, springiness; $p \leq 0.05$ resilience, gumminess), no letter not significant.

Table S21- Texture Profile Analysis data in the five days of storage. Factorial ANOVA for "percentages-method" T4

Sample	Hardness	Springiness	Resilience	Gumminess	Chewiness
Method A					
5%	20.62±6.18d	0.79±0.06c	0.61±0.15c	61.62±17.20d	49.20±19.93c
7.50%	25.60±3.90cd	0.85±0.02bc	0.68±0.04ab	82.87±18.47d	70.12±17.53bc
10%	39.78±8.71ab	0.87±0.02ab	0.63±0.08bc	114.94±12.64bc	101.85±11.04ab
Method B					
5%	27.85±10.56cd	0.90±0.02ab	0.73±0.01a	85.47±14.62cd	72.88±12.41bc
7.50%	45.73±4.88a	0.88±0.05ab	0.72±0.04a	162.52±51.96a	137.93±57.67a
10%	33.68±6.79bc	0.91±0.04a	0.69±0.04a	136.23±19.11ab	111.76±50.97ab

Data are expressed as means ± standard deviations. Different letters indicate ($p \leq 0.001$ hardness, springiness, gumminess; $p \leq 0.01$ resilience; $p \leq 0.05$ chewiness), no letter not significant.

Table S22 - Factorial ANOVA for "Sampling-Percentage"

Sample	Polyphenols (mg GAE/g d.m)	DPPH (mg trolox eq/g d.m)	Water Activity
FAB-5%	0.26±0.04c	0.28±0.05cde	0.88±0.03a
FAB-7.5%	0.32±0.04b	0.34±0.13bcd	0.87±0.01ab
FAB-10%	0.47±0.12a	0.48±0.07a	0.77±0.02c
FAS-5%	0.14±0.01e	0.22±0.06e	0.76±0.03cd
FAS-7.5%	0.21±0.04d	0.35±0.08bc	0.81±0.07bc
FAS-10%	0.27±0.04c	0.50±0.06a	0.83±0.03ab
FAM-5%	0.15±0.02e	0.22±0.09e	0.70±0.08de
FAM-7.5%	0.20±0.06d	0.28±0.12de	0.67±0.01e
FAM-10%	0.27±0.08c	0.40±0.17b	0.69±0.05e

Data are expressed as means ± standard deviations. Different letters indicate $p \leq 0.001$, no letter not significant.

Table S23 - Polyphenols content, antioxidant activity and water activity, in breads with different integration percentages. Factorial ANOVA for "Sampling -Method"

Sample	Polyphenols (mg GAE/g d.m)	DPPH (mg trolox eq/g d.m)	Water Activity
Method A			
FAB	0.40±0.13a	0.44±0.10a	0.83±0.05
FAS	0.18±0.04e	0.41±0.12a	0.80±0.05
FAM	0.25±0.08c	0.41±0.11a	0.70±0.06
Method B			
FAB	0.29±0.07b	0.30±0.10b	0.85±0.06
FAS	0.22±0.07d	0.30±0.13b	0.80±0.06
FAM	0.16±0.03f	0.19±0.05c	0.68±0.05

Data are expressed as means ± standard deviations. Different letters indicate $p \leq 0.001$, no letter not significant.

Table S24 - Polyphenols content, antioxidant activity and water activity, in breads with different integration percentages. Factorial ANOVA for "Percentage-Sampling"

Sample	Polyphenols (mg GAE/g d.m)	DPPH (mg trolox eq/g d.m)	Water Activity
Method A			
5%	0.20±0.07e	0.29±0.03c	0.81±0.05a
7.50%	0.26±0.08c	0.41±0.03b	0.76±0.09ab
10%	0.38±0.16a	0.54±0.00a	0.76±0.09b
Method B			
5%	0.16±0.05f	0.18±0.05d	0.75±0.12b
7.50%	0.23±0.06d	0.23±0.05d	0.80±0.10ab
10%	0.29±0.08b	0.37±0.10b	0.78±0.04ab

Data are expressed as means ± standard deviations. Different letters indicate p≤0.001, no letter not significant.
